# JR-AB2-011 induces fast metabolic changes independent of mTOR complex 2 inhibition in human leukemia cells

**DOI:** 10.1007/s43440-024-00649-7

**Published:** 2024-09-11

**Authors:** Tereza Kořánová, Lukáš Dvořáček, Dana Grebeňová, Kateřina Kuželová

**Affiliations:** Department of Proteomics, Institute of Hematology and Blood Transfusion, U Nemocnice 1, Prague, 128 20 Czech Republic

**Keywords:** Mitochondrial respiration, OCR, Acute myeloid leukemia, AML

## Abstract

**Background:**

The mechanistic target of rapamycin (mTOR) is a crucial regulator of cell metabolic activity. It forms part of several distinct protein complexes, particularly mTORC1 and mTORC2. The lack of specific inhibitors still hampers the attribution of mTOR functions to these complexes. JR-AB2-011 has been reported as a specific mTORC2 inhibitor preventing mTOR binding to RICTOR, a unique component of mTORC2. We aimed to describe the effects of JR-AB2-011 in leukemia/lymphoma cells, where the mTOR pathway is often aberrantly activated.

**Methods:**

The impact of JR-AB2-011 on leukemia/lymphoma cell metabolism was analyzed using the Seahorse platform. AKT phosphorylation at Ser473 was used as a marker of mTORC2 activity. mTOR binding to RICTOR was assessed by co-immunoprecipitation. RICTOR-null cells were derived from the Karpas-299 cell line using CRISPR/Cas9 gene editing.

**Results:**

In leukemia/lymphoma cell lines, JR-AB2-011 induced a rapid drop in the cell respiration rate, which was variably compensated by an increased glycolytic rate. In contrast, an increase in the respiration rate due to JR-AB2-011 treatment was observed in primary leukemia cells. Unexpectedly, JR-AB2-011 did not affect AKT Ser473 phosphorylation. In addition, mTOR did not dissociate from RICTOR in cells treated with JR-AB2-011 under the experimental conditions used in this study. The effect of JR-AB2-011 on cell respiration was retained in RICTOR-null cells.

**Conclusion:**

JR-AB2-011 affects leukemia/lymphoma cell metabolism via a mechanism independent of mTORC2.

**Supplementary Information:**

The online version contains supplementary material available at 10.1007/s43440-024-00649-7.

## Introduction

Mechanistic target of rapamycin (mTOR) is a large (289 kDa) serine/threonine kinase, that integrates various signals from nutrients, growth factors, energy, and oxygen levels. mTOR regulates many aspects of eukaryotic cell behavior such as proliferation, survival, metabolism, and cytoskeleton dynamics. Aberrant activation of mTOR signaling is associated with tumorigenesis in many malignant diseases [1, 2], including hematological tumors [3]. The kinase operates in multiprotein complexes with distinct functions. mTOR complex 1 (mTORC1) and mTOR complex 2 (mTORC2) share the catalytic core, mTOR, as well as the subunits mLST8 and DEPTOR. mTORC1 and mTORC2 can be discriminated by the presence of specific subunits: Regulatory-Associated Protein of mTOR (RAPTOR) and Rapamycin-Insensitive Companion of mTOR (RICTOR), respectively. In addition to the two canonical complexes, a third mTOR complex (mTORC3) has been described but its properties are still largely unknown [4]. mTORC1 and mTORC2 have many distinct roles, but the detailed functional analysis of individual mTOR complexes is hampered by the lack of specific inhibitors [5]. JRAB2-011 has been developed recently for specific targeting of mTORC2 [6].

The Phosphoinositide-3 Kinase (PI3K)-AKT-mTOR signaling pathway is upregulated in 60–80% of patients with acute myeloid leukemia (AML) [7]. Overactivation may occur due to recurrent mutations [8] or via extracellular signals coming from the bone marrow microenvironment, which is known to protect leukemia blasts from chemotherapy [9, 10]. Despite promising preclinical evidence, inhibition of mTORC1 had limited clinical effects due to various compensatory and regulatory feedback loops [11]. Dynamic changes in mTORC1 activity during AML development represent an additional challenge to the establishment of suitable treatment protocols based on mTOR inhibition [12]. To overcome these limitations, combination therapies targeting downstream molecules or parallel pathways are being investigated [7, 13–15]. Dual mTORC1/mTORC2 inhibitors may be used alone or in combination with other drugs [16]. However, global mTOR suppression might have serious side effects, as the AKT-mTOR pathway is required for hematopoiesis, the physiological function of immune cells [17], and normal hematopoietic stem cells under stress conditions [3]. Specific inhibition of mTORC2 has therapeutic potential in AML, as genetic depletion of RICTOR in a mouse model led to overactivation of mTORC1 and exhaustion of leukemia stem cells [18].

Although mTOR is the central regulator of cell metabolism, the roles of the individual mTOR complexes have not been fully elucidated. This is true even for the most commonly studied metabolic processes – oxidative phosphorylation and aerobic glycolysis. mTORC1 promotes glycolysis through the induction of the transcription factors c-MYC and Hypoxia-Inducible Factor 1 (HIF1)-α [5]. mTORC1 activity was also associated with mitochondrial respiration [19, 20]. mTORC2 is involved in the regulation of glycolytic processes through AKT-dependent or AKT-independent mechanisms [5]. The possible role of mTORC2 in oxidative phosphorylation is still poorly understood.

Metabolic processes are also tightly related to immune cell functions. For example, proinflammatory macrophages (M1) depend on glycolysis, whereas the immune-suppressive polarization program of M2 macrophages requires the induction of fatty acid oxidation [21]. JR-AB2-011 inhibited M2 macrophage polarization and prevented the therapeutic effect of dioscin in a mouse model of ulcerative colitis, presumably via mTORC2 inhibition [21]. This finding could be relevant for the treatment of AML, as increased frequencies of M2 macrophages were observed in the bone marrow of patients with AML, and high levels of M2 macrophages were associated with poor disease outcomes [22].

Taken together, many previous studies provided rationale for mTORC2 inhibition in AML and warranted detailed analysis of the respective roles of mTORC1 and mTORC2 in different types of hematopoietic cells. The original aim of this work has been to study the effects of mTORC2 inhibition by JR-AB2-011 on mitochondrial respiration and aerobic glycolysis in leukemia/lymphoma cells. We found, however, that the observed changes in cell metabolism induced by JR-AB2-011 are not due to mTORC2 inhibition.

## Materials and methods

### Chemicals

JR-AB2-011 (MedChemExpress, HY-122022) was supplied by Scintila (Czech Republic) and dissolved in dimethyl sulfoxide (DMSO, Sigma‒Aldrich, #D8418) to make a 10 mM stock solution. A stock solution of rapamycin (Sigma-Aldrich, #553210-5MG) was made at a 5 mM concentration in DMSO. CAL-101 was purchased from SelleckChem (#S2226) and dissolved in DMSO to make a 10 mM stock solution. Aliquots of all inhibitors were kept at -40 °C.

### Cell lines and primary AML cells

The cell lines (Jurkat, Karpas-299, OCI-AML3, MOLM-13, MV4-11, and KG-1) were purchased from the German Collection of Microorganisms and Cell Cultures (Germany). After receipt, the cells were briefly expanded, and aliquots were cryopreserved for later use. The majority of cell lines were cultured in the Roswell Park Memorial Institute (RPMI)-1640 medium with 10% fetal calf serum (FCS), 100 U/mL penicillin, and 100 µg/mL streptomycin at 37 °C in a 5% CO_2_ humidified atmosphere, except for OCI-AML3 (alpha-Minimum Essential Medium, 20% FCS). All cell lines were split three times a week.

Primary cells from the peripheral blood of AML patients with hyperleukocytosis were obtained by leukapheresis before therapy initiation. The basic characteristics of primary samples are given in Supplementary Table S1. The leukapheresis product was diluted 10-fold in phosphate-buffered saline (PBS), and the mononuclear cell fraction was separated using Histopaque-1077 (Sigma‒Aldrich, #H8889). The cells were washed three times in PBS and resuspended in RPMI-1640 medium with 10% FCS and antibiotics (100 U/mL penicillin, 100 µg/mL streptomycin) at 5 × 10^6^/mL. Cell aliquots were treated overnight with JR-AB2-011 and used for metabolic measurements.

#### Ethics statement

All patients included in the study provided their written informed consent to the use of their biological material for research purposes. All experimental procedures concerning primary cells were anonymized. The project was approved by the Ethics Committee of the Institute of Hematology and Blood Transfusion (Prague, Czech Republic) on the 15th of April, 2021 (approval ID 22–13853 S). All procedures followed the ethical standards of the responsible committees on human experimentation (institutional and national) and with the Helsinki Declaration of 1975, as revised in 2008.

### Cell metabolism measurement

CellTak (Corning, #354240) was used to coat Seahorse XFp plates (Agilent, #103022-100). Control or pretreated leukemia cells (5 × 10^4^ cells/well for cell lines, 2 × 10^5^ cells/well for primary cells) were seeded in 50 µL Seahorse medium (Agilent, #103576–100) supplemented with glucose (10 mM), pyruvate (1 mM), and glutamine (2 mM). The plate was briefly centrifuged (1 min, 200 g, gentle brake) and incubated for 20 min at 37 °C without CO_2_. Subsequently, 130 µL of the medium was added, with or without JR-AB2-011 as appropriate. The plate was further incubated for 60 min at 37 °C without CO_2_ and subjected to analysis in the Seahorse XFp device (Agilent). The oxygen consumption rate (OCR) and the extracellular acidification rate (ECAR) were measured using the MitoStress Test kit (Agilent, #103010–100) following the manufacturer´s instructions. The last injection was supplemented with 2-deoxyglucose (2-DG, Sigma, #D8375) to obtain the ECAR background value due to non-glycolytic acidification. If not otherwise indicated, the final well concentrations were as follows: oligomycin (OM) 1 µM, carbonyl cyanide-4 (trifluoromethoxy) phenylhydrazone (FCCP) 0.3 and 0.5 µM, rotenone/antimycin A 0.5 µM, and 2-DG 50 mM.

As illustrated in Fig. S2, the metabolic rates were calculated as follows: the background values (i.e., the last measured points) were subtracted from OCR/ECAR values measured in the 4th cycle (before OM injection) to obtain basal OCR/ECAR. Maximal OCR was obtained by background subtraction from the maximal OCR value measured after FCCP injection. Maximal ECAR was obtained by background subtraction from the ECAR value measured in the 7th cycle (before FCCP injection).

To follow the kinetics of changes induced by JR-AB2-011, the plate with cells was incubated for 45 min at 37 °C without CO_2_, the inhibitor was added directly to the sample wells, and the plate was placed immediately into the Seahorse XFp analyzer. The equilibration step was skipped. No substances were injected during metabolism monitoring.

### Western blot

Protein amounts and phosphorylation status were assessed using standard protocols for western blot. Briefly, the cells were pelleted by centrifugation, washed once with PBS, and lysed for 30 min at 4 °C in modified Radioimmunoprecipitation Assay (RIPA) lysis buffer (50 mM 4-(2-hydroxyethyl)-1-piperazineethanesulfonic acid; 0.15 M NaCl; 2 mM ethylenediaminetetraacetic acid (EDTA); 0.1% nonyl phenoxypolyethoxylethanol (NP-40); 0.05% sodium deoxycholate; 25 mM NaF) with freshly added 1 mM phenylmethylsulfonyl fluoride (PMSF), protease inhibitors (P8340-1ML, Sigma-Aldrich), and phosphatase inhibitors (P5726-1ML, Sigma-Aldrich). Cellular debris was removed by centrifugation (16,000 g/4°C/15 min), and the lysate was mixed 1:1 (v/v) with 2x Laemmli sample buffer and incubated for 5 min at 95 °C. An equivalent of 20 µg total protein was resolved in a polyacrylamide gel and transferred onto a membrane using TransBlot Turbo (Bio-Rad). The membrane was blocked in Tris-buffered saline (TBS) with 3% bovine serum albumin and 0.1% Tween-20 (TBST) for 1 h and then incubated with the primary antibody diluted in TBST for 1.5 h at room temperature (for mTOR and RICTOR N-term only) or overnight at 5 °C (all other antibodies). Then, the membrane was washed six times in TBST and incubated with horseradish peroxidase-conjugated secondary antibody for 1 h. The Clarity Western Enhanced Chemiluminescence Substrate (Bio-Rad, #170–5060) was used to induce a chemiluminescence signal, which was detected using G: BOX iChemi XT-4 (Syngene). The GeneTools 4.3.7.0 software was employed for signal quantification. The antibodies used were the following: 4E-BP1 (Cell Signaling, #9452), p4E-BP1 Ser65 (Cell Signaling, #13443), AKT (Cell Signaling, #4691), pAKT Ser473 (Cell Signaling, #4060), mTOR (ProteinTech, #66-888-1-Ig), RICTOR (Santa Cruz, sc-81538), RICTOR N-term (ProteinTech, #27248-1-AP), and βACTIN (Santa Cruz, sc-47778).

### Co-immunoprecipitation

The cells (10^7^ per sample) were washed twice with ice-cold PBS and lysed for 30 min on the ice at constant agitation in 1 mL lysis buffer (100 mM NaCl; 1% NP-40; 50 mM Tris-HCl, pH 7.5; 5 mM EDTA) with freshly added 1 mM PMSF, protease inhibitors (P8340-1ML, Sigma-Aldrich) and phosphatase inhibitors (P5726-1ML, Sigma-Aldrich). The lysate was centrifuged (10,000 g/30 min/4°C), and 100 µL of supernatant was mixed 1:1 with 2x Laemmli sample buffer and boiled for 5 min (input sample). The rest of the supernatant was incubated with either mTOR (ProteinTech, #66-888-1-Ig) or control IgG antibody (Santa Cruz, sc-2025) for 2 h at 4 °C. Subsequently, Protein A/G PLUS Agarose beads (Santa Cruz, sc-2003) were added, and the sample was further incubated for 2 h at 4 °C. The suspension was washed 4 times with washing buffer (100 mM NaCl; 0.1% NP-40; 50 mM Tris-HCl, pH 7.5; 5 mM EDTA; freshly added 1 mM PMSF; protease and phosphatase inhibitors), and the pellets were resuspended in 2x Laemmli sample buffer and boiled for 10 min. Finally, the sample was centrifuged (10,000 g/2 min), and the supernatant was aspirated, yielding the immunoprecipitate sample. Samples were analyzed by western blotting for changes in mTOR and RICTOR protein levels using the antibodies specified in the previous paragraph.

### Gene knockout

*RICTOR* knockout was generated in the Karpas-299 cell line via nonhomologous end joining (NHEJ) using the Clustered Regularly Interspaced Short Palindromic Repeats (CRISPR)/Cas9 system and guide RNA (gRNA) with the following sequence: AAGAGAGATTCTCCAAAATG. The Cas9 enzyme, gRNA, and electroporation enhancer were obtained from Integrated DNA Technologies (Alt-R™ S.p. HiFi Cas9 Nuclease V3 #1081061, Alt-R^®^ CRISPR‒Cas9 tracr/crRNA #1072534, Alt-R^®^ Cas9 Electroporation Enhancer #1075916). Karpas-299 cells (1 × 10^5^) were transfected using the Invitrogen™ Neon^®^ Transfection System (1300 V, 10 ms, 3 cycles, 10 µL Kit, buffer R, 25 pmol Cas9 enzyme, 25 pmol crRNA: tracrRNA duplex). After transfection, Karpas-299 cells were seeded in 24-well plates and cultured for 3 days. To obtain stable RICTOR-null clones, the cell suspension was diluted to 5 cells/mL, and 100 µL aliquots were distributed into a 96-well culture plate. Cell growth was regularly monitored by visual inspection. Wells containing a single colony were selected for further cell expansion and analysis. *RICTOR* knockout status was then checked by western blotting.

### Statistical analyses

Statistical evaluation of the experimental results was performed using GraphPad Prism version 9.5.0 (GraphPad Software, San Diego, California, USA). The p-value limit for statistically significant differences between groups was set to 0.05. Shapiro-Wilke test was used to check the normal distribution of the data that were subsequently analyzed using parametric tests. Differences in metabolic rates between control and treated samples were evaluated using a two-tailed paired Student´s t-test. Western blot data were normalized to ACTIN and to values obtained from untreated samples (100%). The values from treated samples were then evaluated using a one-sample Student’s t-test in comparison with the control value (100%). All the graphs presented in this work were created using the GraphPad Prism software.

When relevant, the statistical significance of differences between groups is indicated by asterisks in the plots: ns = not significant, *p < 0.05, **p < 0.01, ***p < 0.001, ****p < 0.0001.

## Results

### Effects of JR-AB2-011 on cell proliferation and viability

JR-AB2-011 doses were preselected based on literature data: 5–10 µM JR-AB2-011 [6]. Under our experimental conditions, cell viability and proliferation were not compromised by 24 h JR-AB2-011 treatment (Supplementary Fig. S1). Fig. S1 also indicates that JR-AB2-011 had only little impact on the cell growth and viability up to 50 µM concentration for up to 48 h treatment.

### Time course of metabolic changes induced by JR-AB2-011

**Fig. 1:**
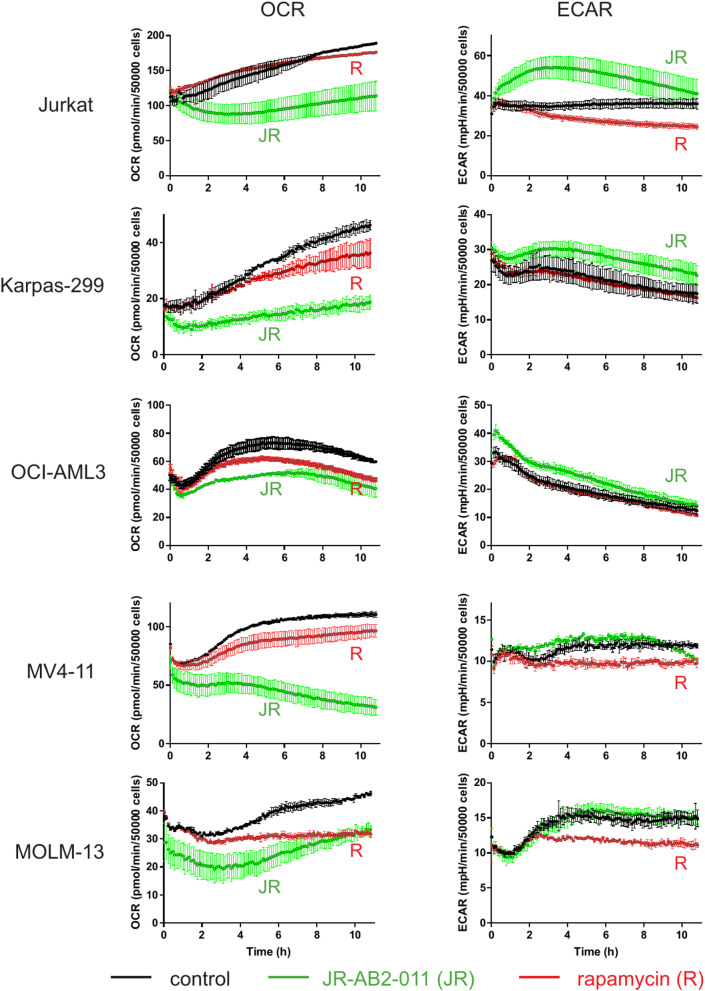
Time course of metabolic changes induced by JR-AB2-011 in myeloid/lymphoid cell lines. The oxygen consumption rate (OCR, left column) and extracellular acidification rate (ECAR, right column) were monitored in real-time using the Seahorse platform, immediately after cell treatment with JR-AB2-011 (5 µM). Rapamycin (50 nM) was used for comparison as an established mTOR inhibitor. The plots show mean ± SD of sample duplicates. Black: untreated cells, green: JR-AB2-011 (JR), red: rapamycin (R)

To gain insight into the kinetics of metabolic changes induced by JR-AB2-011, we first monitored cell metabolism in real-time using the Seahorse platform. Figure 1 shows representative records of the cell respiration rate (measured as the oxygen consumption rate, OCR) and glycolysis rate (measured as the extracellular acidification rate, ECAR) for leukemia/lymphoma cell lines. Rapamycin was used for comparison as an established mTOR inhibitor. In general, JR-AB2-011 treatment (green lines) induced a drop in OCR, which was sometimes compensated by increased ECAR. The metabolic changes following cell treatment with JR-AB2-011 occurred rapidly compared to those induced by rapamycin (red lines). Based on these results, the preincubation time of cell lines with JR-AB2-011 was fixed to 1 h. Standard Seahorse analyses were subsequently performed using 1 h and 24 h incubation times.

### Effect of JR-AB2-011 on cell metabolic rates

**Fig. 2:**
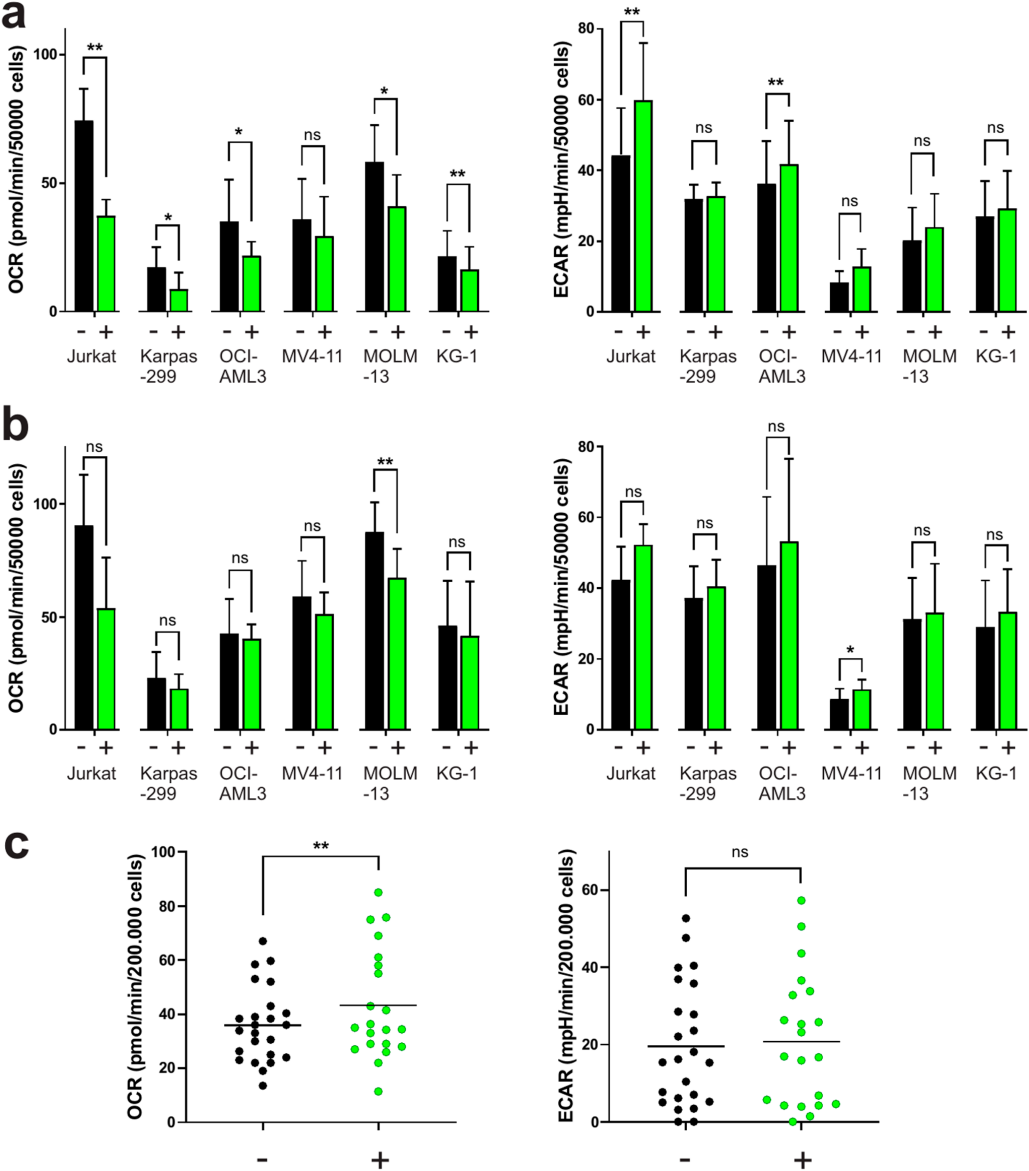
Impact of JR-AB2-011 on the basal metabolic rates in leukemia/lymphoma cells. The basal rates of cell respiration (OCR, left) and glycolysis (ECAR, right) were measured using the Seahorse platform. Representative Seahorse records are shown in Supplementary Fig. S2. **a**: Cell lines were treated for 1 h with 5 µM JR-AB2-011 during incubation in a Seahorse plate. Results are expressed as mean ± SD of 3 to 5 independent experiments for each cell line. **b**: Cell lines were treated for 24 h with 5 µM JR-AB2-011 under standard culture conditions and then seeded into a Seahorse plate. Results are expressed as mean ± SD of 3 to 6 independent experiments for each cell line. **c**: Primary cells from patients with AML were treated for 24 h with 10 µM JR-AB2-011 and seeded into a Seahorse plate. Results from 21 different individuals with AML at diagnosis. See the Supplementary Table S1 for the basic characteristics of primary samples. Differences in OCR/ECAR between treated cells (green) and the corresponding controls (black) were evaluated using two-tailed paired Student´s t-test

Examples of standard Seahorse records are shown in Supplementary Fig. S2. Metabolic rates (OCR and ECAR) were calculated from Seahorse records as described in the Methods section and illustrated in Fig. S2. In preliminary tests, JR-AB2-011 at 5 or 10 µM concentration had similar effects on metabolic rates (Supplementary Fig. S3), and we thus used 5 µM JR-AB2-011 for cell line treatment in all subsequent experiments (Fig. 2). In agreement with the results of real-time monitoring (Fig. 1), basal OCR was reduced after 1 h treatment with JR-AB2-011 (Fig. 2a). The differences in OCR, resp. ECAR values between treated samples and the corresponding untreated controls were evaluated using a paired Student´s t-test with the following results for 1 h treatment: Jurkat OCR t_3_ = 7.797, *p* = 0.0044; Jurkat ECAR t_3_ = 8.395, *p* = 0.0035; Karpas-299 OCR t_3_ = 4.654, *p* = 0.0187; Karpas-299 ECAR t_4_ = 0.9826, *p* = 0.3815; OCI-AML3 OCR t_5_ = 2.999, *p* = 0.0301; OCI-AML3 ECAR t_5_ = 3.629, *p* = 0.0151; MV4-11 OCR t_2_ = 3.348, *p* = 0.0788; MV4-11 ECAR t_2_ = 2.897, *p* = 0.1014; MOLM-13 OCR t_3_ = 4.854, *p* = 0.0167; MOLM-13 ECAR t_3_ = 1.491, *p* = 0.2326; KG-1 OCR t_3_ = 8.253, *p* = 0.0037; KG-1 ECAR t_3_ = 1.449, *p* = 0.2431. The effects of JR-AB2-011 partially persisted for 24 h following the cell line treatment (Fig. 2b), but the differences did not retain the statistical significance in the majority of comparisons: Jurkat OCR t_2_ = 3.946, *p* = 0.0586; Jurkat ECAR t_2_ = 1.543, *p* = 0.2627; Karpas-299 OCR t_4_ = 1.176, *p* = 0.3064; Karpas-299 ECAR t_4_ = 1.718, *p* = 0.1610; OCI-AML3 OCR t_3_ = 0.2617, *p* = 0.8105; OCI-AML3 ECAR t_3_ = 0.8169, *p* = 0.4738; MV4-11 OCR t_2_ = 1.169, *p* = 0.3628; MV4-11 ECAR t_2_ = 5.095, *p* = 0.0364; MOLM-13 OCR t_3_ = 6.94, *p* = 0.0061; MOLM-13 ECAR t_3_ = 0.8716, *p* = 0.4476; KG-1 OCR t_3_ = 1.289, *p* = 0.2877; KG-1 ECAR t_3_ = 1.52, *p* = 0.2259.

Similar analyses were also performed with primary cells isolated from the peripheral blood of patients with AML (Fig. 2c). In a few pilot experiments, we did not observe any impact of JR-AB2-011 after 1 h of treatment and we thus focused on 24 h incubation. As larger cell numbers were required to obtain sufficiently high Seahorse signals from primary cells, twice as high doses of OM and FCCP were used. In contrast to cell lines, an increase in OCR was often induced by JR-AB2-011 in primary cells. The differences in OCR, resp. ECAR values between treated samples and the corresponding untreated controls were evaluated using a paired Student´s t-test with the following results: OCR t_20_ = 2.922, *p* = 0.0084; ECAR t_20_ = 1.977, *p* = 0.0620.

### Effect of JR-AB2-011 on glucose uptake

The rate of glucose uptake was measured in the cell lines using a fluorescent glucose analog, 2-NBDG. As shown in Fig. S4, samples treated for 1.5 h with JR-AB2-011 tended to accumulate higher amounts of glucose, but the differences were not statistically significant.

### Changes in phosphorylation of mTOR downstream targets

**Fig. 3:**
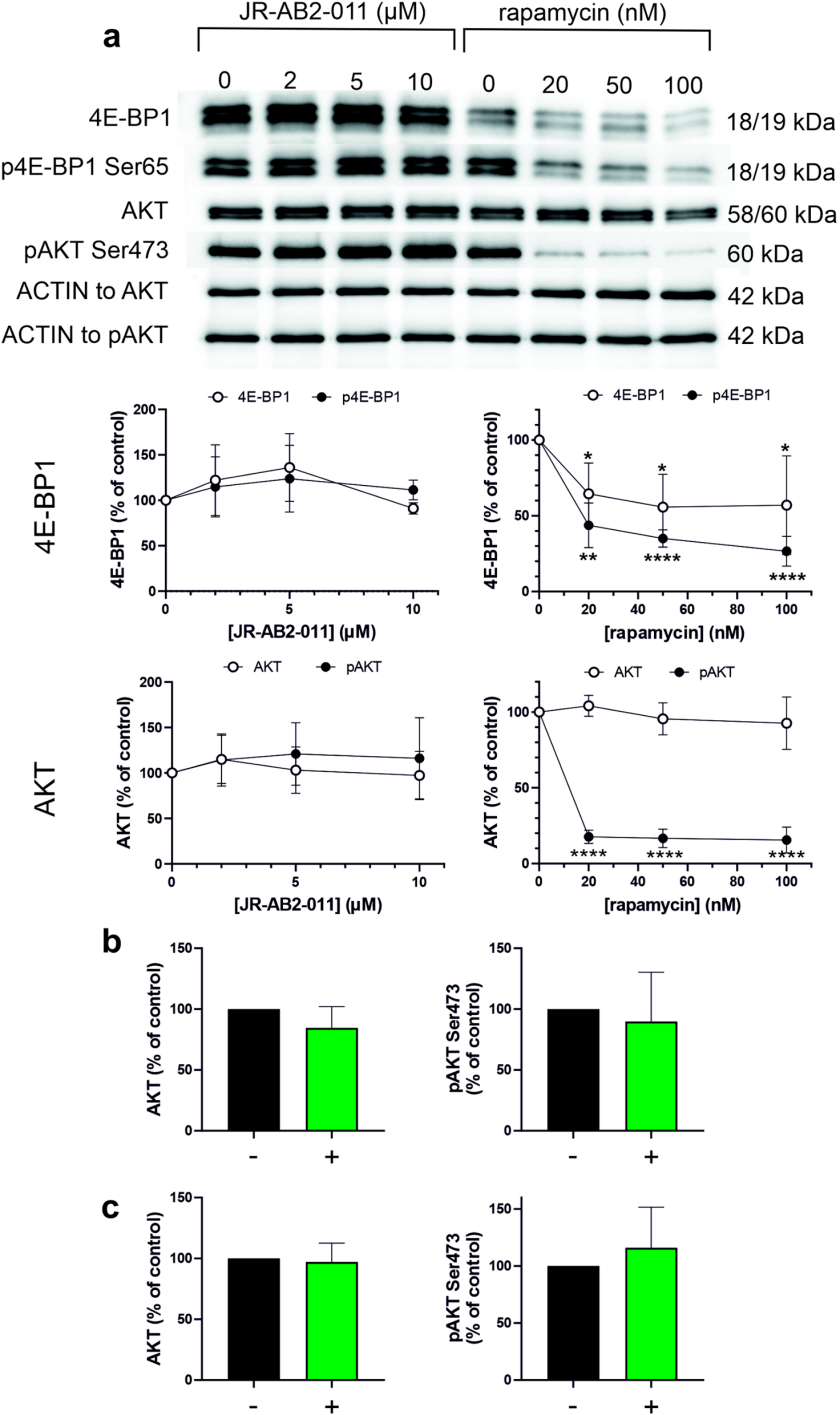
Impact of JR-AB2-011 on 4E-BP1 and AKT phosphorylation in Jurkat cells. Rapamycin was used as a positive control. Cell lysates from control and treated cells were analyzed by western blot. The membranes containing cell proteins were cut in two. The upper parts were incubated with total AKT or pAKT Ser473 antibody and the lower parts with total 4E-BP1 or p4E-BP1 Ser65 antibody, respectively. ACTIN was reprobed after AKT/pAKT analysis in the upper parts. **a**: Cells were treated for 1 h with JR-AB2-011 (left) or for 24 h with rapamycin (right) at increasing concentrations as indicated and harvested for western blot analysis. Top: representative western blot images. Bottom: relative band intensities normalized to ACTIN and the corresponding untreated controls (100%). Results are expressed as mean ± SD of values from repeated experiments: *N* = 3, resp. 5 for 4E-BP1 analysis in cells treated with JR-AB2-011, resp. rapamycin; *N* = 7, resp. 6 for AKT analysis in cells treated with JR-AB2-011, resp. rapamycin. One-sample Student´s t-test was used to compare the data from treated samples with the control value (100%). **b**: Effect of higher JR-AB2-011 dose on AKT Ser473 phosphorylation. AKT and pAKT Ser473 levels in untreated cells and cells treated for 1 h with 50 µM JR-AB2-011 were determined by western blot. The band intensities were normalized to ACTIN and to the corresponding untreated controls (black bars, 100%). Results are expressed as mean ± SD of values from 4 repeated experiments. **c**: Effect of longer JR-AB2-011 treatment on AKT Ser473 phosphorylation. AKT and pAKT Ser473 levels in untreated cells and cells treated for 24 h with 5 µM JR-AB2-011 were determined by western blot. The band intensities were normalized to ACTIN and to the corresponding untreated controls (black bars, 100%). Results are expressed as mean ± SD of values from 3 repeated experiments

To check for JR-AB2-011 efficiency at the doses used, we analyzed changes in phosphorylation occurring at 4E-BP1 and AKT, downstream targets of mTORC1 and mTORC2, respectively (Fig. 3). Rapamycin was used as a positive control in Jurkat cells. Both 4E-BP1 Ser65 and AKT Ser473 were indeed less phosphorylated after 24 h rapamycin treatment (Fig. 3a). Normalized protein expression in Jurkat cells treated with rapamycin (rap) at different concentrations was tested using one-sample Student´s t-test for comparison with the control (untreated cells, 100%). The resulting statistical values were the following: AKT rap 20 nM t_5_ = 1.471, *p* = 0.2014; rap 50 nM t_5_ = 1.048, *p* = 0.3428; rap 100 nM t_5_ = 1.040, *p* = 0.3460; pAKT Ser473 rap 20 nM t_5_ = 45.71, *p* < 0.0001; rap 50 nM t_5_ = 33.53, *p* < 0.0001; rap 100 nM t_5_ = 24.14, *p* < 0.0001; 4E-BP1 rap 20 nM t_4_ = 3.887, *p* = 0.0177; rap 50 nM t_4_ = 4.551, *p* = 0.0104; rap 100 nM t_4_ = 2.953, *p* = 0.0419; p4E-BP1 Ser65 rap 20 nM t_4_ = 8.537, *p* = 0.001; rap 50 nM t_4_ = 25.5, *p* < 0.0001; rap 100 nM t_4_ = 16.73, *p* < 0.0001.

Intriguingly, no decrease in pAKT Ser473 was detected after Jurkat cell treatment with JR-AB2-011 (Fig. 3a). No effect of JR-AB2-011 on AKT phosphorylation was detected even at higher inhibitor doses (Fig. 3b) or with longer treatment time (Fig. 3c). To further confirm the specificity of pAKT Ser473 antibody, we treated cell lines with the PI3K/mTOR inhibitor CAL-101. As expected, the signal was largely reduced in this positive control in all cell lines tested (Supplementary Fig. S5).

### Co-immunoprecipitation mTOR/RICTOR

**Fig. 4:**
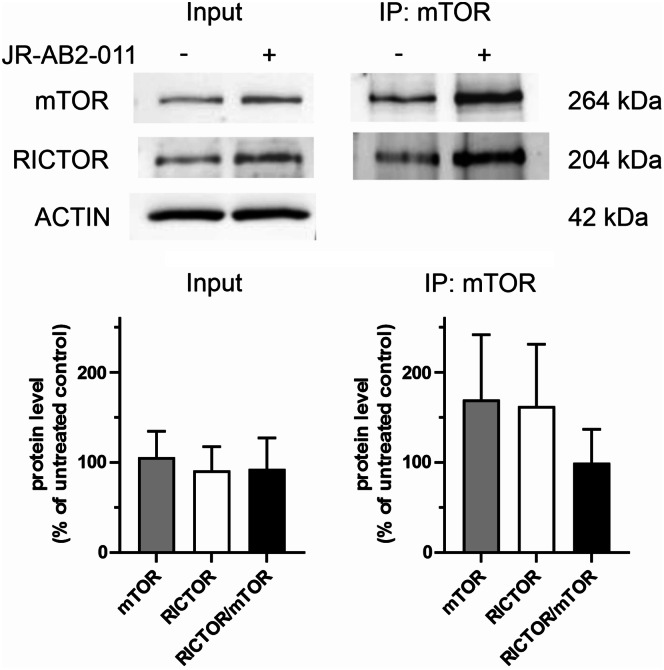
Impact of JR-AB2-011 on mTOR interaction with RICTOR assessed by co-immunoprecipitation. Karpas-299 cells were treated for 1 h with 5 µM JR-AB2-011, and mTOR complexes were isolated using agarose beads with anti-mTOR antibody. RICTOR (pray) and mTOR (bait) amounts in the input cell lysates (Input, positive control) and precipitated samples (IP: mTOR) were quantified using western blot. Band intensities were normalized to ACTIN in the Input samples. The untreated samples (control) were taken as 100% for each membrane. Top: representative western blot membranes. Bottom: mean ± SD of values from 6 independent experiments. No RICTOR signal was detected in immunoprecipitates with a control IgG antibody (negative control shown in Supplementary Fig. S6)

To check for the expected mTOR dissociation from RICTOR after 1 h cell treatment with JR-AB2-011, we performed co-immunoprecipitation experiments with the Karpas-299 cell line, which was reported to have constitutively active mTOR [23]. RICTOR co-precipitated with mTOR in all samples, and the RICTOR/mTOR ratio in the immunoprecipitates was not affected by JR-AB2-011 (Fig. 4): one-sample Student´s t-test comparing the RICTOR/mTOR values from samples treated with JR-AB2-011 to the control value (100%) yielded t_5_ = 0.01106, *p* = 0.9916. No RICTOR signal was detected in negative control samples where the precipitation beads were coated with nonspecific IgG instead of the mTOR antibody (Supplementary Fig. S6).

### Metabolic changes induced by JR-AB2-011 treatment in RICTOR-null cells

**Fig. 5:**
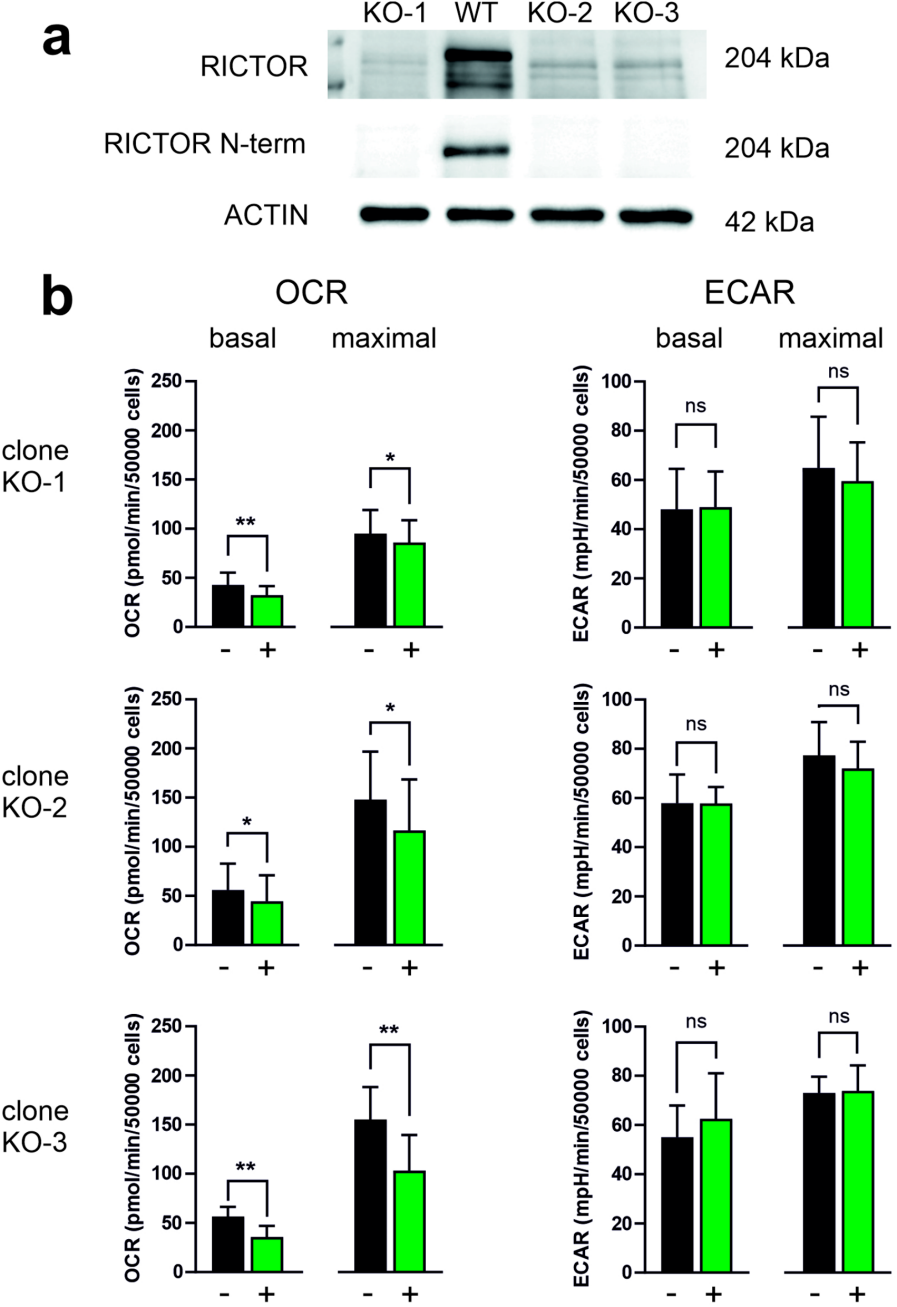
Impact of JR-AB2-011 on the metabolic rates in RICTOR-null cells. Three clones of Karpas-299 cells with *RICTOR* knockout were obtained and further tested. a: Western blot analysis of RICTOR levels in clones with *RICTOR* knockout (KO-1, KO-2, KO-3) compared to parental cells (WT). Two different antibodies were used to confirm the absence of RICTOR at the protein level. ACTIN was used as the loading control. b: Metabolic rates in RICTOR-null cells treated for 1 h with 5 µM JR-AB2-011. Results are expressed as mean ± SD of values from 8, 5, resp. 3 repeated experiments for the clones KO-1, KO-2, and KO-3. The differences between treated cells and untreated controls were evaluated using two-tailed paired Student´s t-test

These surprising observations led us to test the effect of JR-AB2-011 in RICTOR-null cells. We performed *RICTOR* knockout in Karpas-299 cells by the CRISPR/Cas9 technique. Three clones lacking RICTOR at the protein level were obtained and further analyzed. The absence of RICTOR was confirmed using two antibodies recognizing different epitopes (Fig. 5a). In all three clones with *RICTOR* knockout, JR-AB2-011 significantly reduced the cell respiration rate (Fig. 5b). The differences in OCR, resp. ECAR values between treated samples and the corresponding untreated controls were evaluated using a paired Student´s t-test with the following results: clone KO-1 OCR basal t_7_ = 4.312, *p* = 0.0035; OCR maximal t_7_ = 2.646, *p* = 0.0331; ECAR basal t_7_ = 0.5619, *p* = 0.5917; ECAR maximal t_7_ = 2.563, *p* = 0.0374; clone KO-2 OCR basal t_4_ = 3.072, *p* = 0.0372; OCR maximal t_4_ = 4.018, *p* = 0.0159; ECAR basal t_4_ = 0.0054, *p* = 0.996; ECAR maximal t_4_ = 1.509, *p* = 0.2059; clone KO-3 OCR basal t_2_ = 23.86, *p* = 0.0018; OCR maximal t_2_ = 25.87, *p* = 0.0015; ECAR basal t_2_ = 2.045, *p* = 0.1775; ECAR maximal t_2_ = 0.3787, *p* = 0.7413. Our attempts to obtain clones with *RICTOR* knockout from Jurkat cells were not successful.

### Effect of JR-AB2-011 in glutamine-free medium

AML cell lines are known to depend on amino acids as a fuel for the Krebs cycle. We thus tested the effects of JR-AB2-011 in a glutamine-free medium. The cells were cultured overnight in the absence of glutamine before treatment with JR-AB2-011. As shown in Supplementary Fig. S7, Jurkat cells displayed a similar response as in the complete medium. In contrast, glutamine starvation of AML cells largely reduced OCR in itself and JR-AB2-011 treatment had no additional effect.

## Discussion

In this work, we described the effects of a novel mTORC2 inhibitor, JR-AB2-011, on cell respiration and glycolytic activity in a panel of myeloid/lymphoid cell lines and leukemia primary cells. JR-AB2-011 induced rapid changes in cell metabolism (Figs. 1 and 2) without inducing cell death (Fig. S1). However, we also provided evidence that JR-AB2-011 did not inhibit mTORC2 under our experimental conditions: no decrease in AKT phosphorylation at Ser473 was detected by western blot (Fig. 3) and no change in RICTOR association with mTOR was observed in co-immunoprecipitation experiments (Fig. 4). In addition, the effect of JR-AB2-011 on cell respiration rate was retained in three different cell clones with RICTOR knock-out (Fig. 5).

We expected JR-AB2-011 could be efficient in myeloid and lymphoid cells as the PI3K/AKT/mTOR pathway is often upregulated in hematological malignancies. Jurkat cells were used for analysis of mTOR signaling in several previous studies, and rapamycin was shown to inhibit both mTORC1 and mTORC2 in this cell line [24]. In Karpas-299 cells, mTOR is reportedly constitutively activated by the fusion kinase NPM-ALK [23]. These two lymphoid cell lines were complemented with a set of AML cell lines. Increasing evidence points to an association between metabolic preferences and clinically relevant properties of AML blasts such as resistance to chemotherapy and immune evasion. On the one hand, a metabolic shift to aerobic glycolysis, known as the Warburg effect, was observed in some AML subtypes and linked to worse prognosis [25]. On the other hand, unlike healthy hematopoietic stem cells, AML stem cells largely depend on oxidative phosphorylation and are metabolically more rigid than bulk AML cells [26]. Interestingly, chemoresistant leukemic cells have almost exclusively high oxidative phosphorylation status [27].

In the cell lines included in this study, JR-AB2-011 induced a drop in the cell respiration rate (OCR), variably compensated by an increase in the glycolysis rate (ECAR), particularly in Jurkat cells (Fig. 2a). These metabolic changes occurred rapidly after JR-AB2-011 addition (Fig. 1) indicating a direct and rather long-lasting effect. It is worth noting that the cells were not under optimal conditions during continuous monitoring (e.g. due to serum starvation, repeated mixing, etc.) and the results presented in Fig. 1 are thus less reliable for longer times. However, no important difference between 1 h and 24 h treatment was detected using standard metabolic measurements (cf. panels a and b in Fig. 2).

In contrast to cell lines, JR-AB2-011 induced an increase in OCR in primary AML cells (Fig. 2c). This observation may reflect metabolic differences between early leukemic progenitors on the one hand and more differentiated bulk leukemia cells on the other hand. In fact, leukemia cell lines are mostly derived from early progenitors whereas bulk leukemia cells prevail in leukapheresis products.

Intriguingly, we did not detect changes in downstream mTOR signaling after JR-AB2-011 treatment. Rapamycin was used as a positive control for Jurkat cells. In agreement with a previous study [24], we observed a large reduction in 4E-BP1 Ser65 and AKT Ser473 phosphorylation, indicating both mTORC1 and mTORC2 inhibition upon Jurkat cell treatment with rapamycin (Fig. 3a). Similarly, AKT phosphorylation was suppressed in AML cell lines treated with CAL-101 (Supplementary Fig. S5). Despite these positive controls, we did not observe any effect of JR-AB2-011 on AKT Ser473 phosphorylation under our experimental conditions (Fig. 3).

JR-AB2-011 was shown to disrupt mTOR binding to RICTOR and suppress AKT phosphorylation in glioblastoma cells after 24 h of treatment [6]. In mouse xenograft models, JR-AB2-011 reduced the growth of glioblastoma tumors [6] and blocked the M2 polarization program in macrophages [28]. However, as documented by our co-immunoprecipitation experiments, RICTOR binding to mTOR was not prevented by 1 h of JR-AB2-011 treatment in Karpas-299 cells (Fig. 4). Higher doses might be required in leukemia cells to inhibit mTOR binding to RICTOR, but we did not detect any decrease in AKT pSer473 levels following treatment of Jurkat cells with up to 50 µM JR-AB2-011 (Fig. 3b). No changes in pAKT Ser473 were detected even after 24 h of incubation with JR-AB2-011 (Fig. 3c). Although JR-AB2-011 may disrupt mTOR association with RICTOR in other cell types [6] or at higher doses [29], it was inefficient as a mTORC2 inhibitor in our model systems.

Furthermore, metabolic analysis of cells with *RICTOR* knockout confirmed that the rapid decrease in OCR induced by JR-AB2-011 occurred even in the absence of RICTOR (Fig. 5). In addition, similar metabolic changes were also found in KG-1 cells (Fig. 2a), which have undetectable pAKT Ser473 levels. The activity of mTORC2 in these cells is thus supposedly very low.

Taken together, our results show that JR-AB2-011 induces rapid metabolic changes independent of mTORC2 inhibition in myeloid/lymphoid cells. Possible unspecific effects of this compound should be taken into account in future studies with other cell types.

## Electronic supplementary material

Below is the link to the electronic supplementary material.

Supplementary Material 1

Supplementary Material 2

Supplementary Material 3

Supplementary Material 4

Supplementary Material 5

Supplementary Material 6

Supplementary Material 7

Supplementary Material 8

Supplementary Material 9

Supplementary Material 10

Supplementary Material 11

Supplementary Material 12

Supplementary Material 13

Supplementary Material 14

Supplementary Material 15

Supplementary Material 16

Supplementary Material 17

Supplementary Material 18

Supplementary Material 19

Supplementary Material 20

Supplementary Material 21

## Data Availability

The datasets generated and/or analyzed during the current study are available from the corresponding author upon reasonable request.
