# Supplementary material for: JR-AB2-011 induces fast metabolic changes independent of mTOR complex 2 inhibition in human leukemia cells
Source: Pharmacol Rep. 2024 Sep 11;76(6):1390–402. doi: 10.1007/s43440-024-00649-7 (PMC11582178; doi:10.1007/s43440-024-00649-7)

JR-AB2-011 induces fast metabolic changes independent of mTOR complex 2 inhibition in human leukemia cells

T.Kořánová, L.Dvořáček, D.Grebeňová, K.Kuželová

Supplementary Figures S1 to S7

Fig.S1: Impact of JR-AB2-011 on cell proliferation and viability.

a. Cell lines were seeded at 5×10^5/ml and treated for 24h with 5 µM JR-AB2-011. Viable cell density (left) and cell viability (right) were determined from sample aliquots mixed with Trypan blue using the TC10 cell counter (BioRad). Mean ± SD of values from 4 to 11 independent experiments for each cell line. Differences between control and treated cells were evaluated using paired Student´s t-test (t_6=3.94, p=0.0076 for MOLM-13; ns for other cell lines).

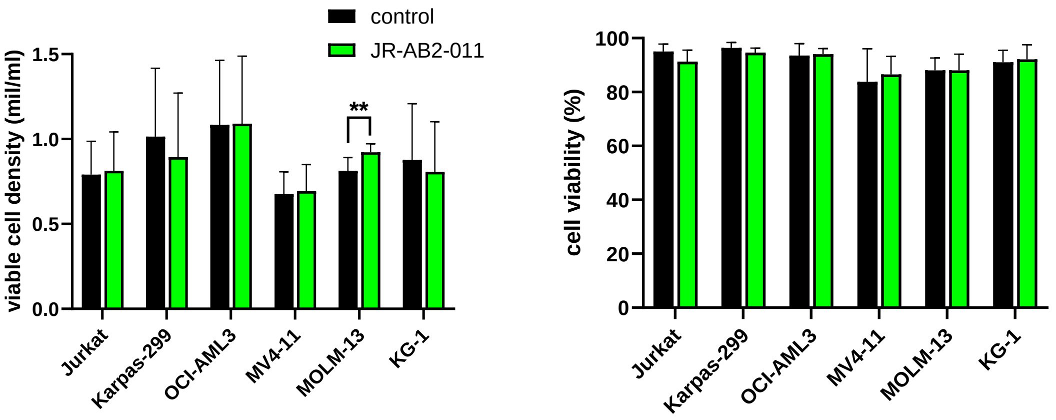

b. Cell lines were seeded at 5×10^5/ml and treated with 5 to 50 µM JR-AB2-011. Viable cell density and cell viability were determined using the TC10 cell counter as above. Cell viability was analyzed in parallel by flow cytometry (propidium iodide exclusion test).

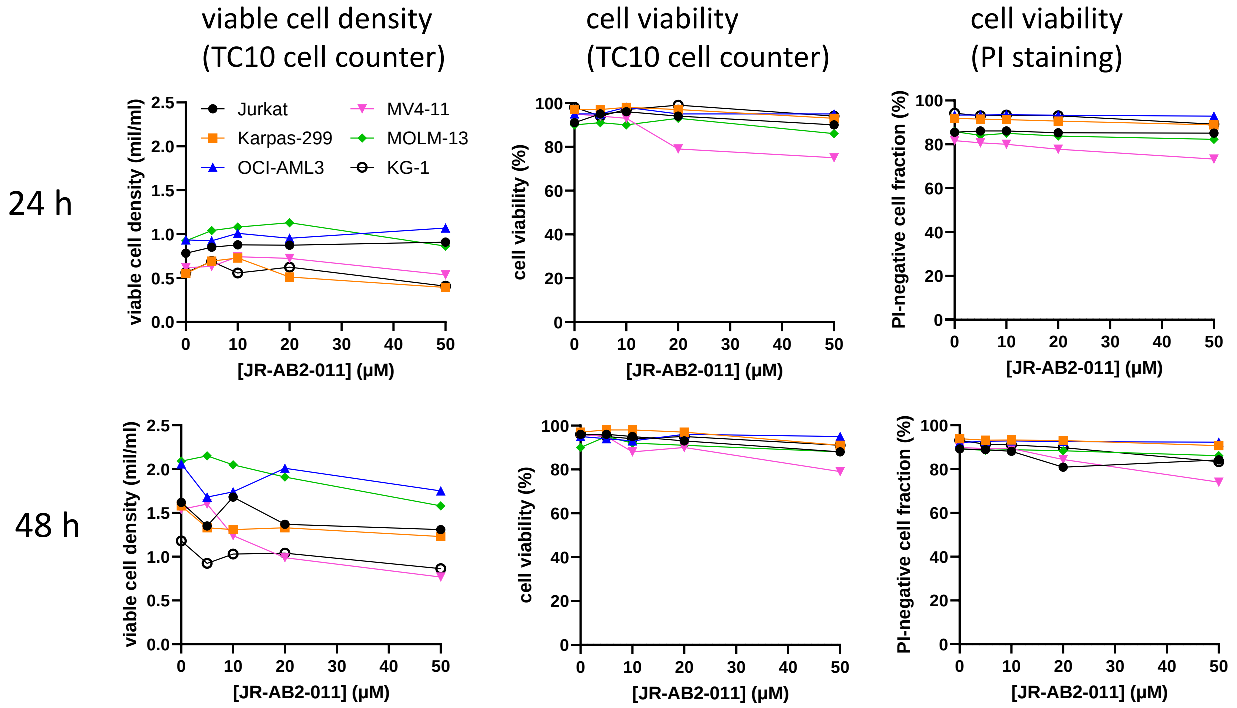

Fig.S2: Examples of Seahorse records and definition of metabolic rates.

Cells were seeded into a Seahorse plate coated with CellTak. The metabolic rates were measured using a Seahorse XFp device. The points represent means ± SD of duplicate wells. Injections of oligomycin (OM), FCCP (two injections) and blocking compounds (rot/AA+2-DG) are indicated by blue arrows. The background values were taken from the last recorded points. The basal and maximal OCR and ECAR levels were calculated as it is illustrated for the control sample (black circles).
In this example, Jurkat cells (50,000/well) were treated in a Seahorse plate for 1 h with JR-AB2-011 5 µM (blue squares) or 10 µM (green triangles).

Oxygen consumption rate (OCR)

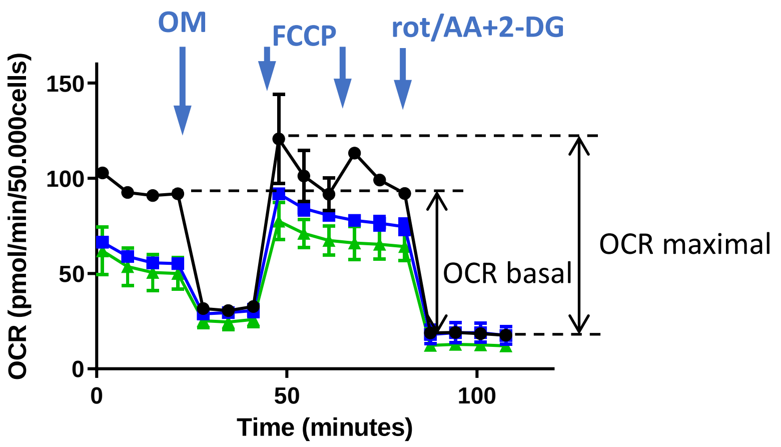

Extracellular acidification rate (ECAR)

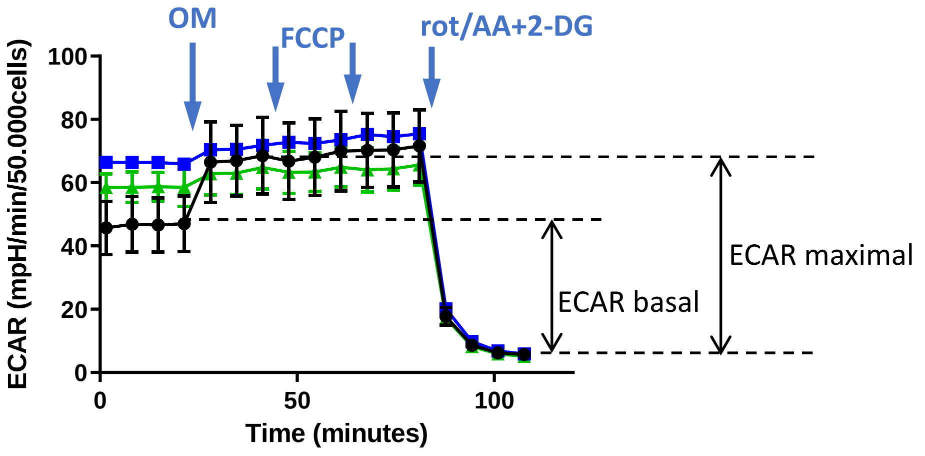

Fig.S3: Effect of higher JR-AB2-011 dose on cell line metabolism

Cells were seeded into a Seahorse plate and treated for 1 h with 5 or 10 µM JR-AB2-011 in dublets. The graphs show the basal metabolic rates (OCR and ECAR) from all performed experiments. Mean ± SD is shown for Karpas-299 and MV4-11 cells where the experiments were repeated twice.

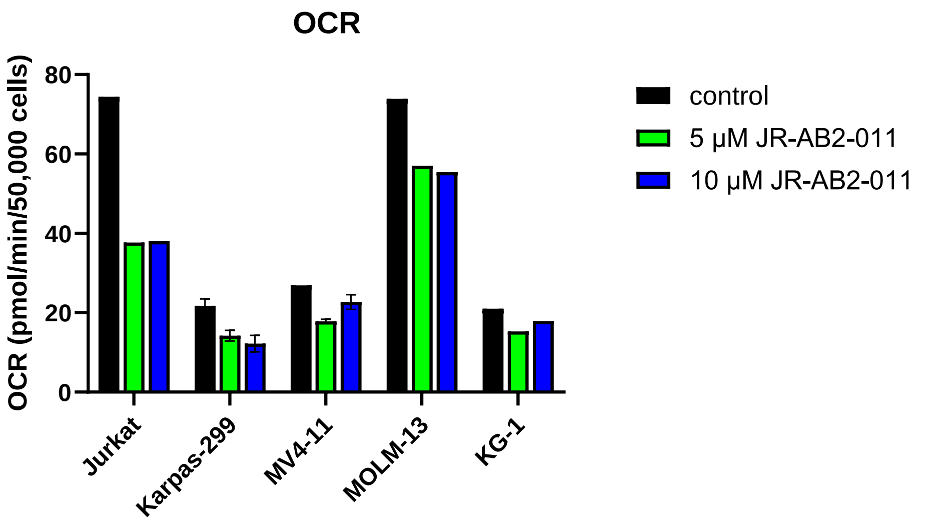

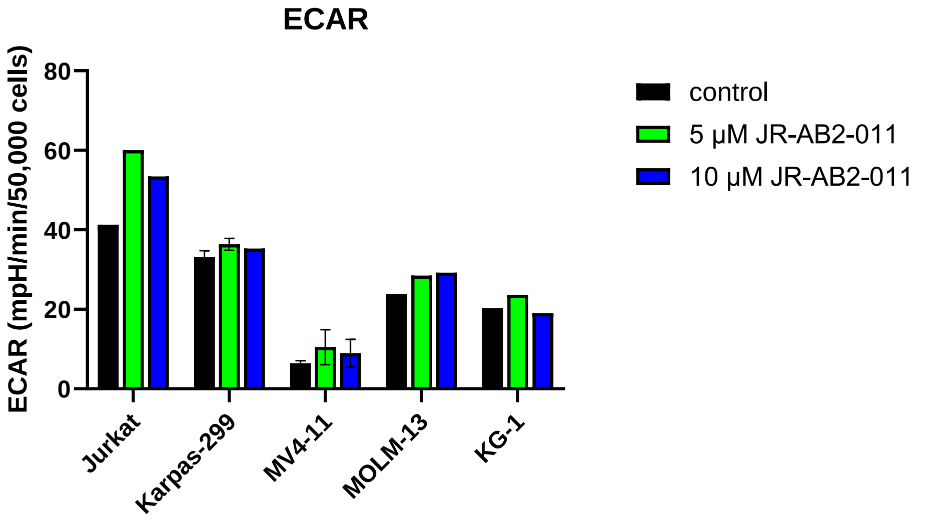

Fig.S4: Effect of JR-AB2-011 on glucose uptake

Cells were pretreated for 30 min with 10 µM JR-AB2-011, then 20 µM 2-NBDG was added for 1 h. The cells were washed and analyzed on a BD Fortessa flow cytometer. The mean fluorescence intensity (MFI) in the FITC channel was determined from 20,000 viable cells per sample. The graph shows mean ± SD of MFI values from 4 to 5 independent experiments for each cell line.

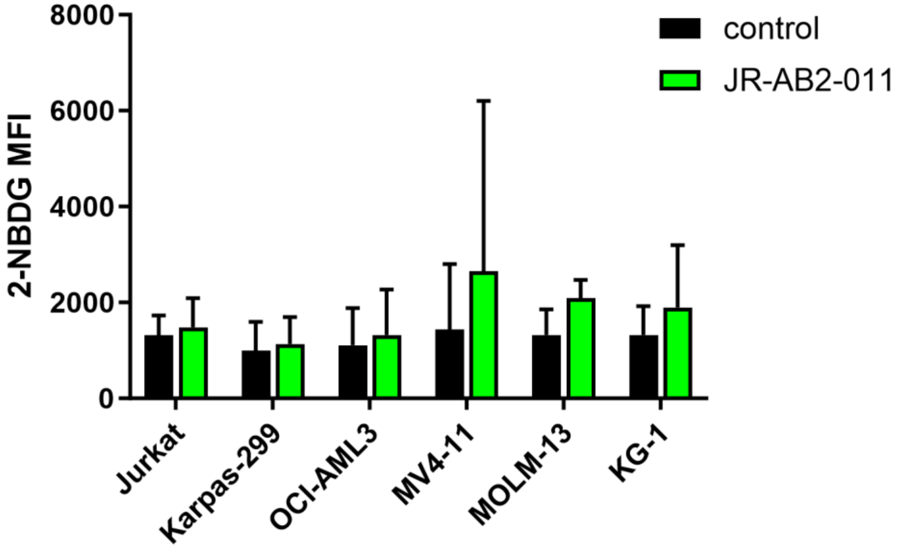

Fig.S5: Effect of CAL-101 as a positive control for AKT Ser473 antibody.

Cell lines were treated for 3 h with 10 µM CAL-101 and harvested for western blot. Total AKT protein amount and AKT phosphorylation at Ser473 were assessed using the corresponding antibodies.

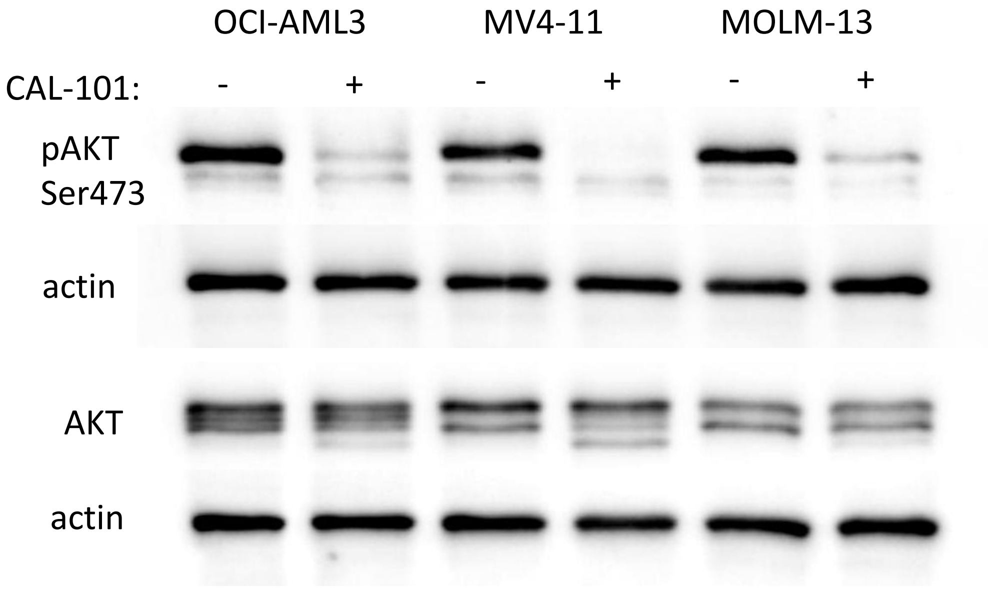

Fig.S6: Co-immunoprecipitation with the control IgG antibody.

Karpas-299 cells were treated for 1 h with 5 µM JR-AB2-011 and harvested for immunoprecipitation (IP) using beads coated with mTOR antibody or with a control IgG antibody. The precipitated proteins were analyzed by western blot (WB) using mTOR or RICTOR antibodies. No signal from mTOR or RICTOR at the correct molecular weight (blue arrows) was detected in the sample with the control IgG.

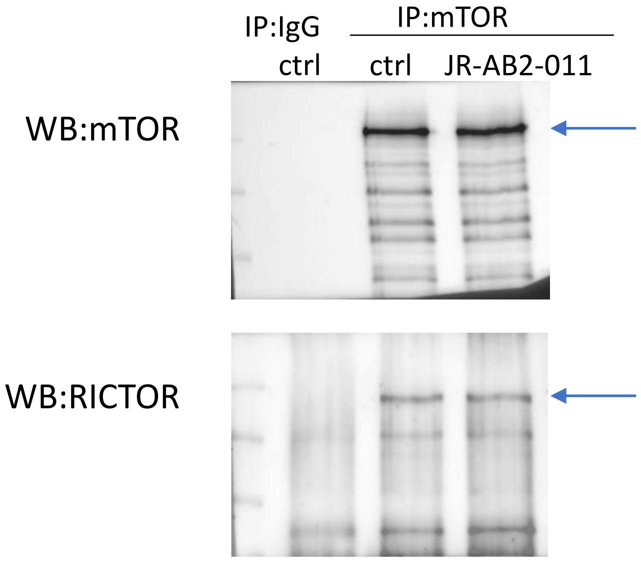

Fig.S7: Effect of JR-AB2-011 under glutamine (Gln) starvation

Cells were starved in the Gln-free medium overnight, then treated for 1 h with 5 µM JR-AB2-011 and analyzed using Seahorse XFp.
Left: Metabolic rates in the complete medium (with Gln), as reported in Fig. 2a.
Right: Metabolic rates obtained in the Gln-free medium. All other conditions were identical to those in Fig. 2a. Performed 3 times for Jurkat and once for Karpas-299, OCI-AML3, and MOLM-13 cells. Treated cells in green.

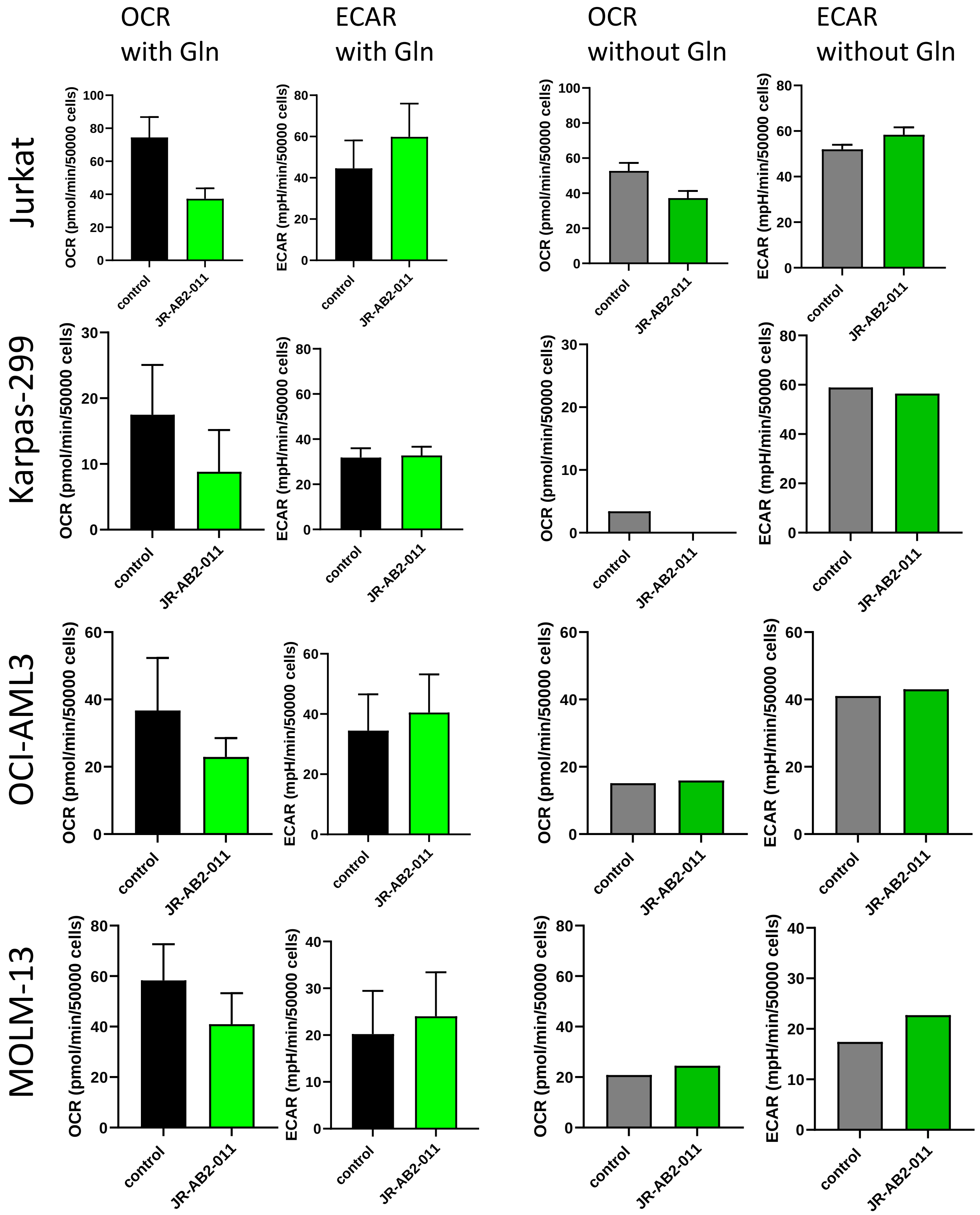

Supplement: Supplementary file 1 — Supplementary Material 1 [file 43440_2024_649_MOESM1_ESM.pdf]
