# Supplementary material for: JR-AB2-011 induces fast metabolic changes independent of mTOR complex 2 inhibition in human leukemia cells
Source: Pharmacol Rep. 2024 Sep 11;76(6):1390–402. doi: 10.1007/s43440-024-00649-7 (PMC11582178; doi:10.1007/s43440-024-00649-7)
Supplement: Supplementary file 3 — Supplementary Material 3 [file 43440_2024_649_MOESM3_ESM.pptx]

## Slide 1
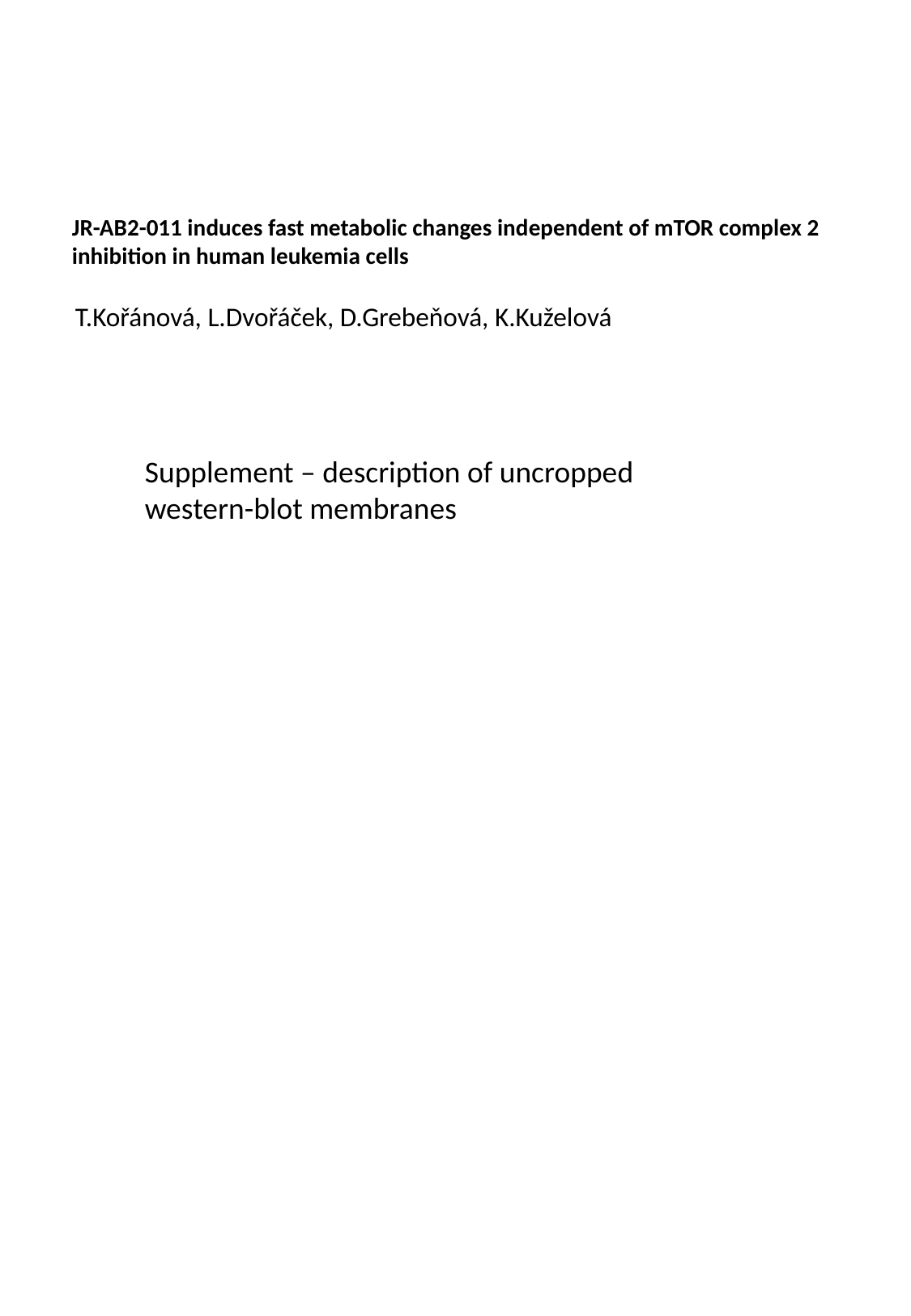

JR-AB2-011 induces fast metabolic changes independent of mTOR complex 2 inhibition in human leukemia cells
T.Kořánová, L.Dvořáček, D.Grebeňová, K.Kuželová
Supplement – description of uncropped
western-blot membranes

## Slide 2
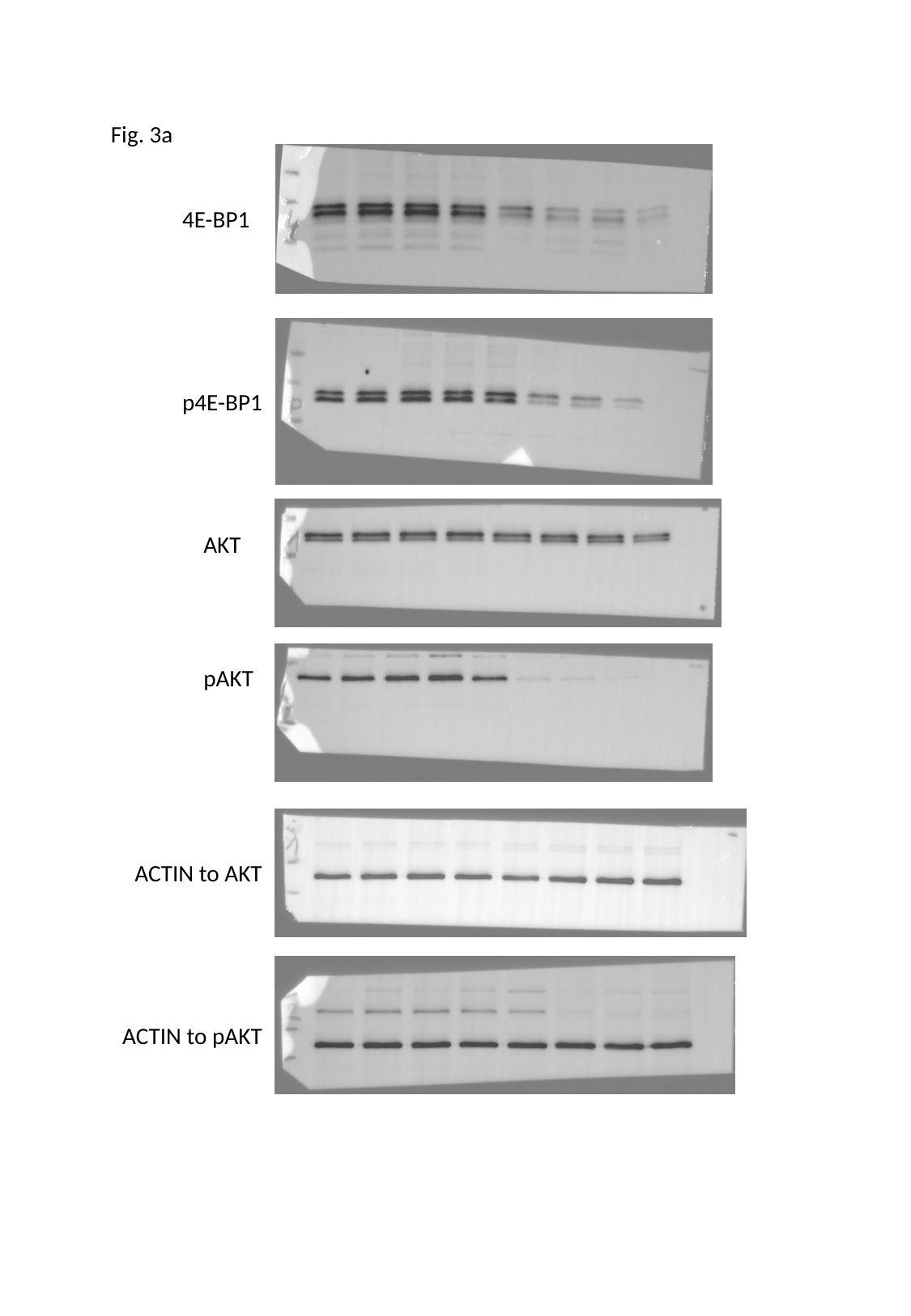

Fig. 3a
4E-BP1
p4E-BP1
AKT
pAKT
ACTIN to AKT
ACTIN to pAKT

## Slide 3
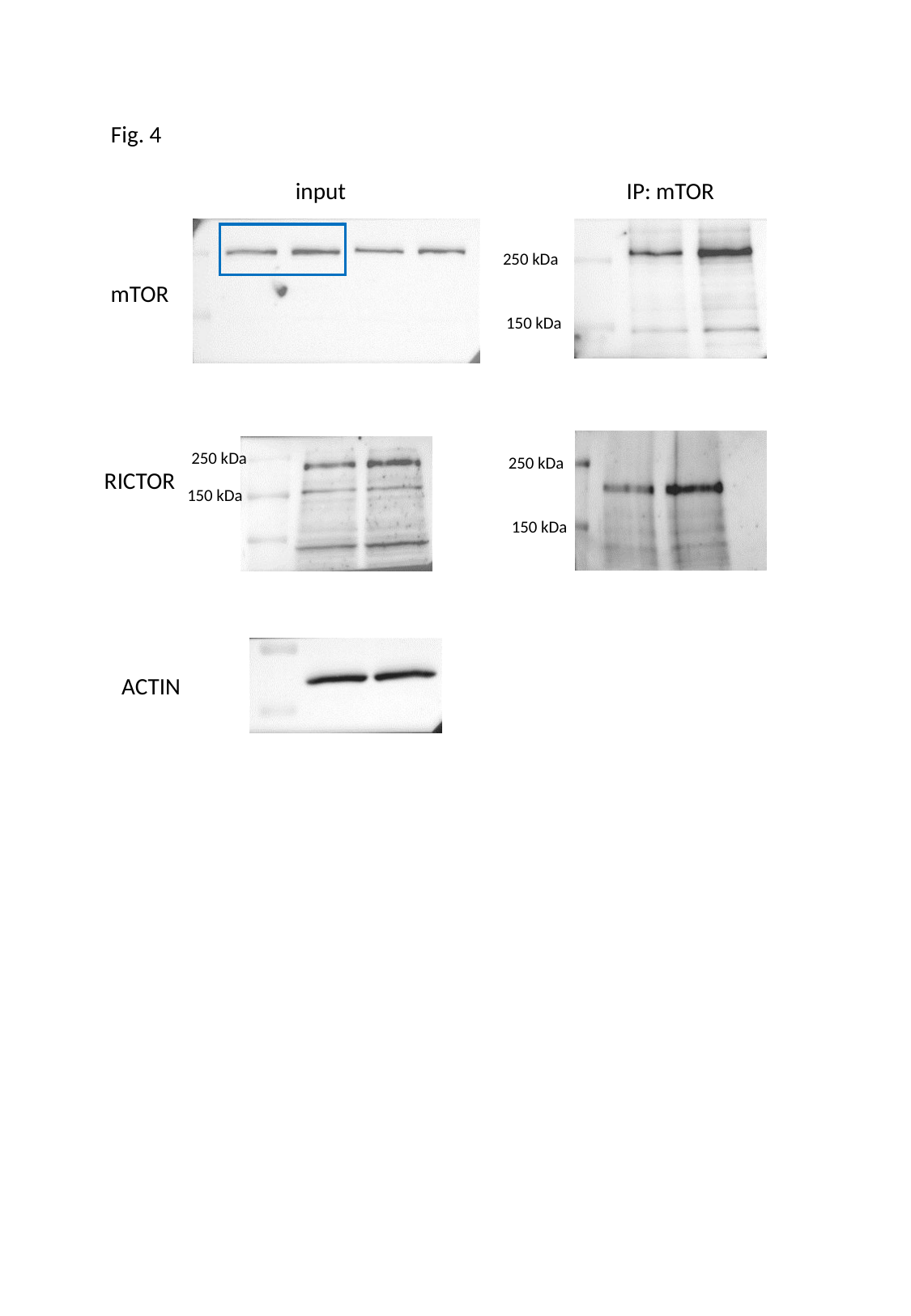

Fig. 4
input
IP: mTOR
250 kDa
mTOR
150 kDa
250 kDa
250 kDa
RICTOR
150 kDa
150 kDa
ACTIN

## Slide 4
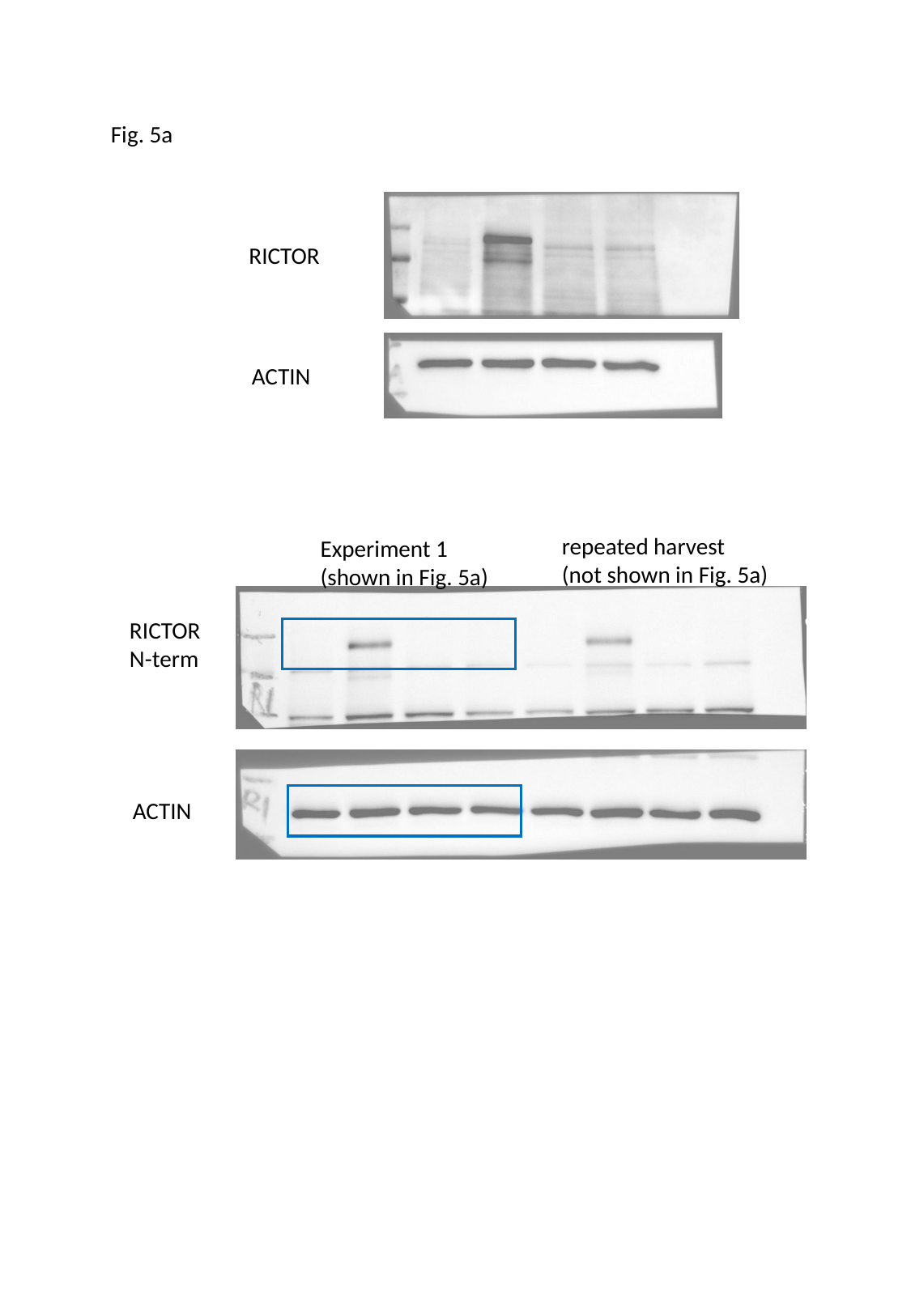

Fig. 5a
RICTOR
ACTIN
repeated harvest
(not shown in Fig. 5a)
Experiment 1
(shown in Fig. 5a)
RICTOR
N-term
ACTIN

## Slide 5
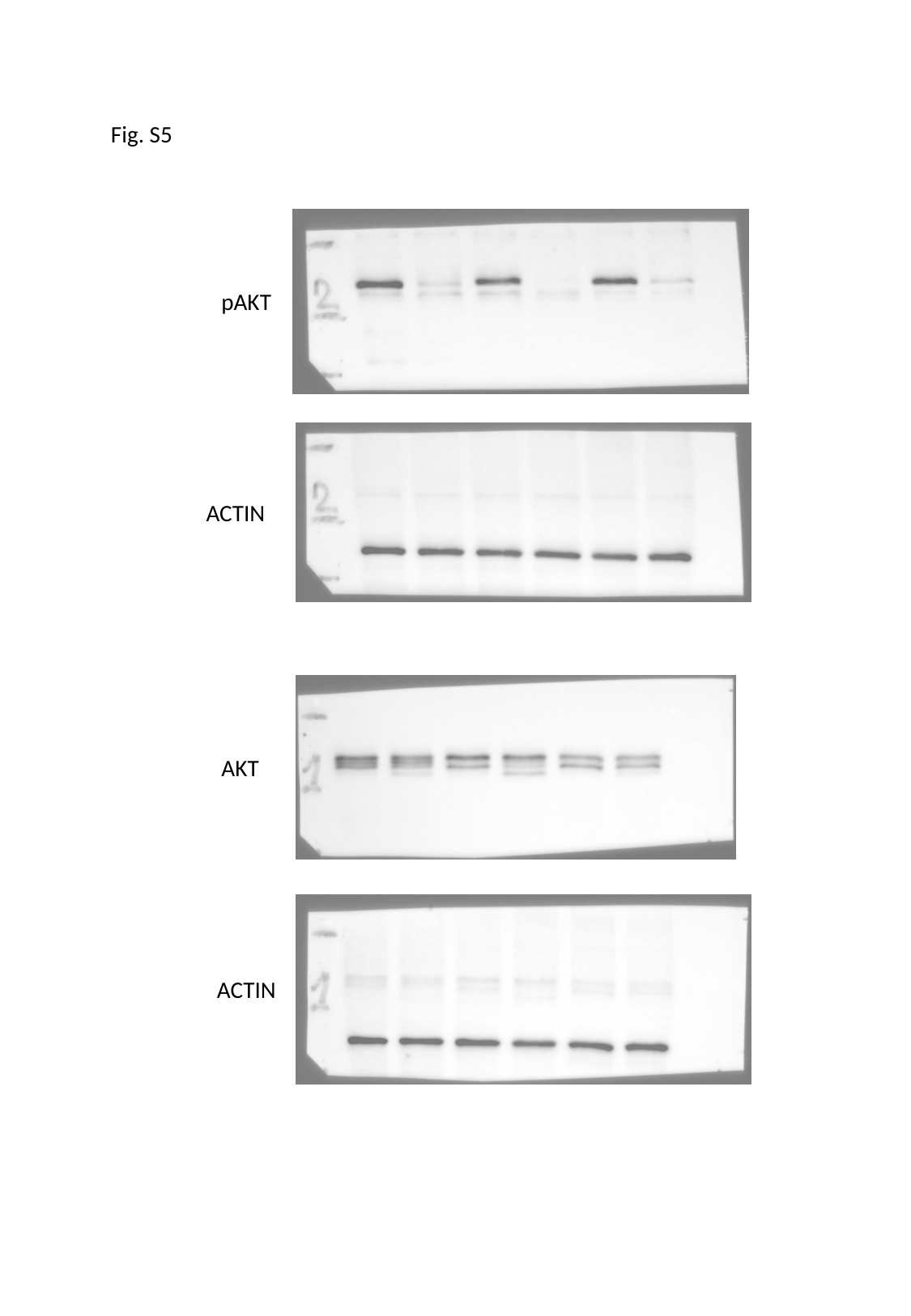

Fig. S5
pAKT
ACTIN
AKT
ACTIN
