# Supplementary figures and images for: JR-AB2-011 induces fast metabolic changes independent of mTOR complex 2 inhibition in human leukemia cells
Source: Pharmacol Rep. 2024 Sep 11;76(6):1390–402. doi: 10.1007/s43440-024-00649-7 (PMC11582178; doi:10.1007/s43440-024-00649-7)

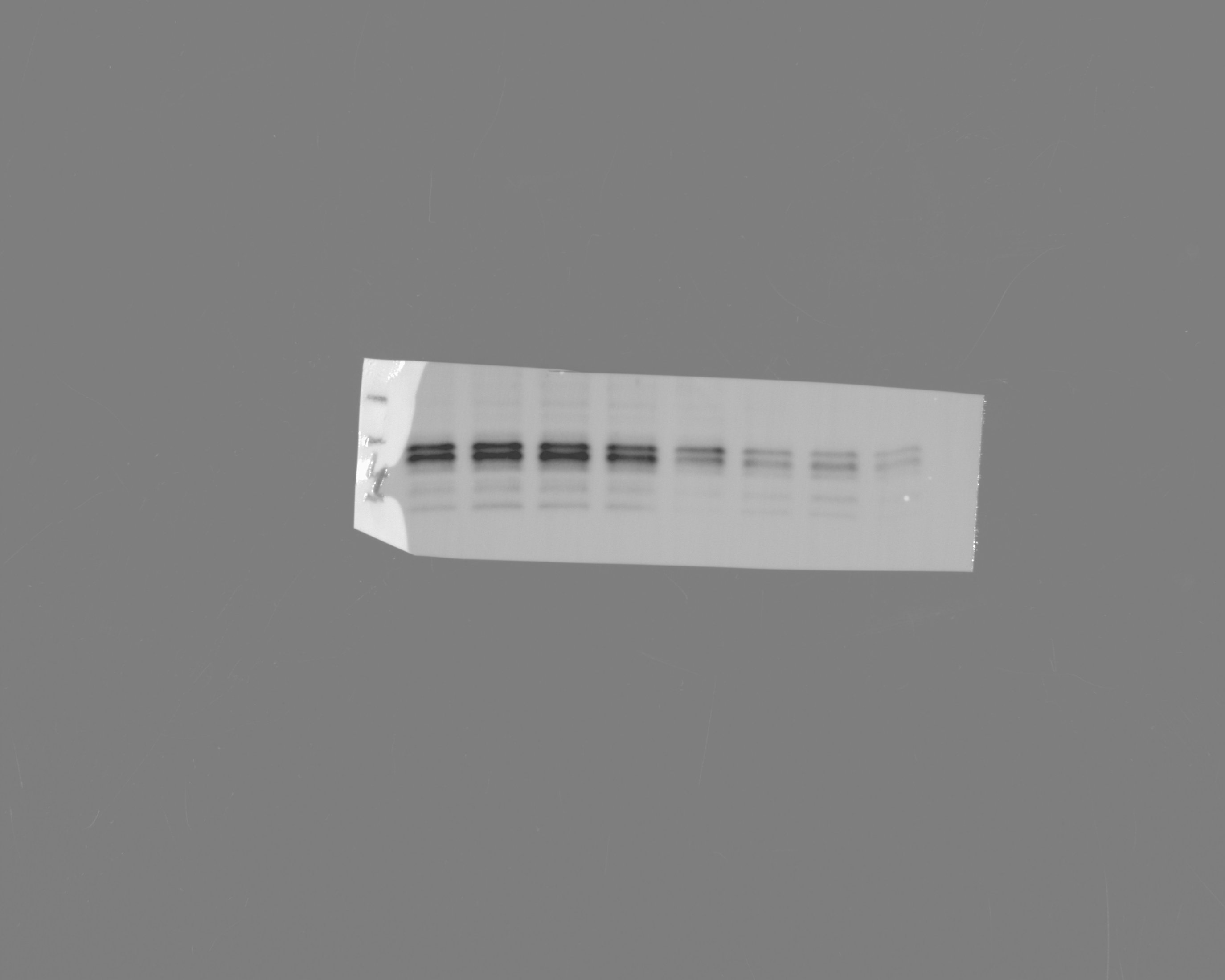

Supplement: Supplementary file 4 — Supplementary Material 4 [file 43440_2024_649_MOESM4_ESM.jpg]

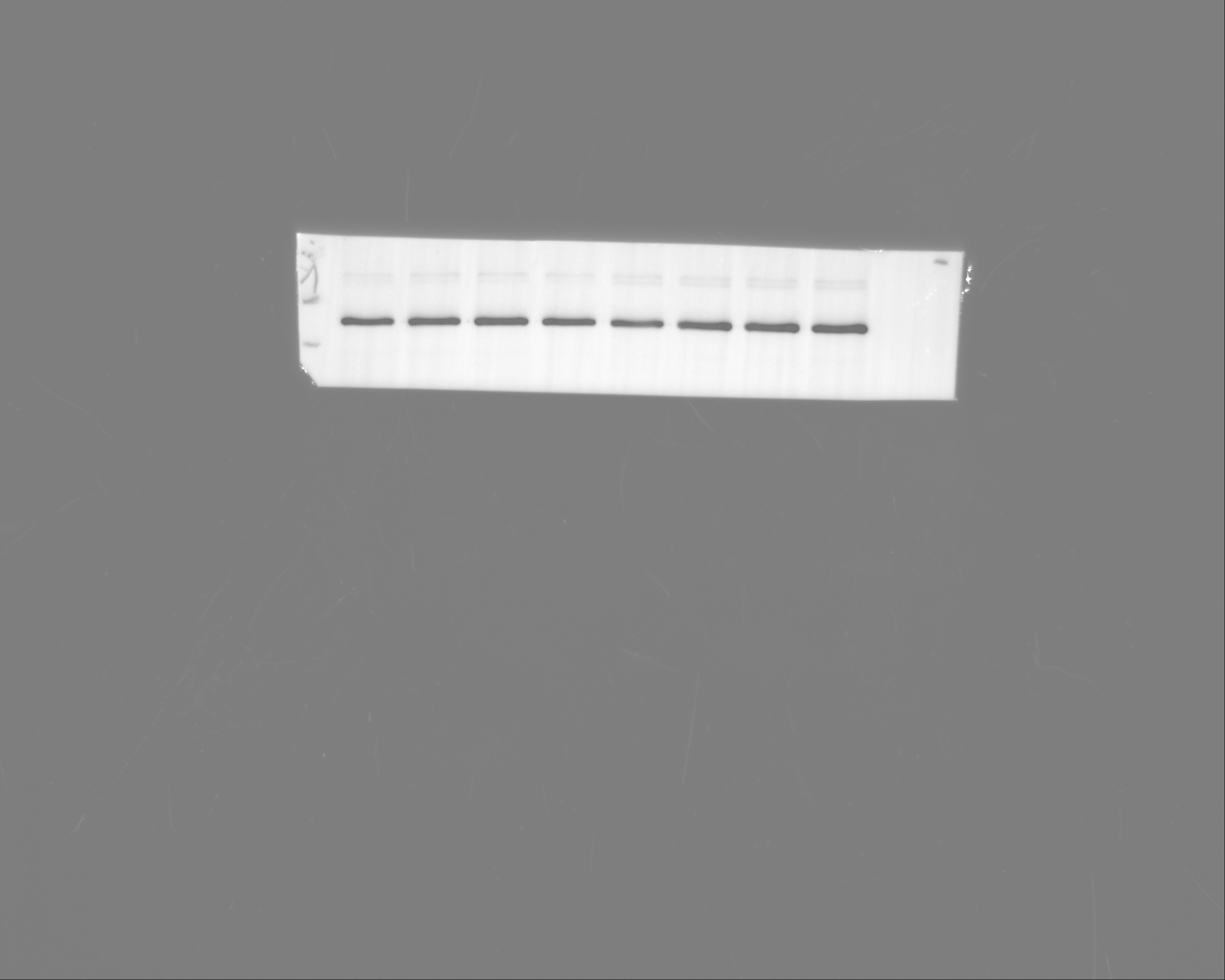

Supplement: Supplementary file 5 — Supplementary Material 5 [file 43440_2024_649_MOESM5_ESM.jpg]

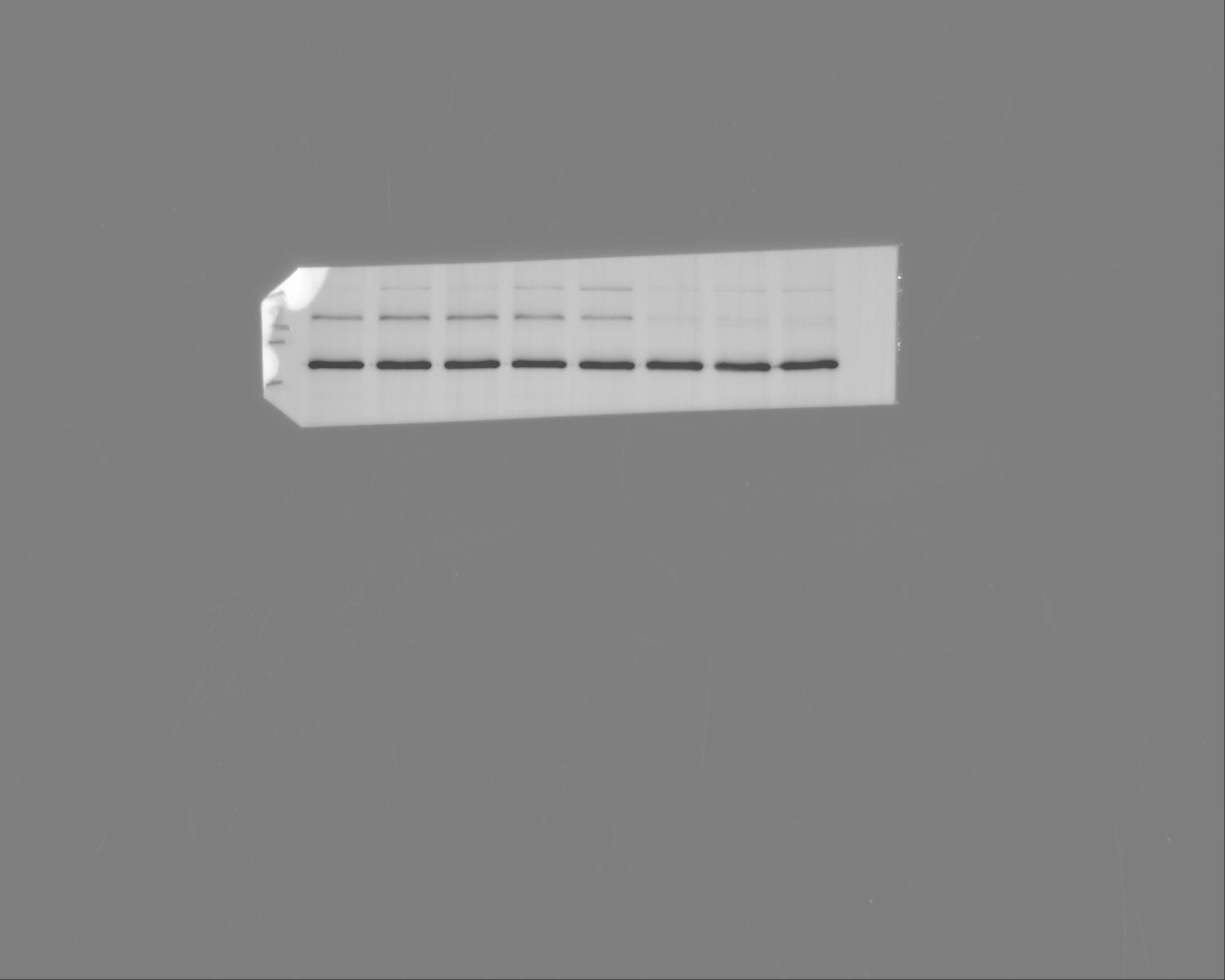

Supplement: Supplementary file 6 — Supplementary Material 6 [file 43440_2024_649_MOESM6_ESM.jpg]

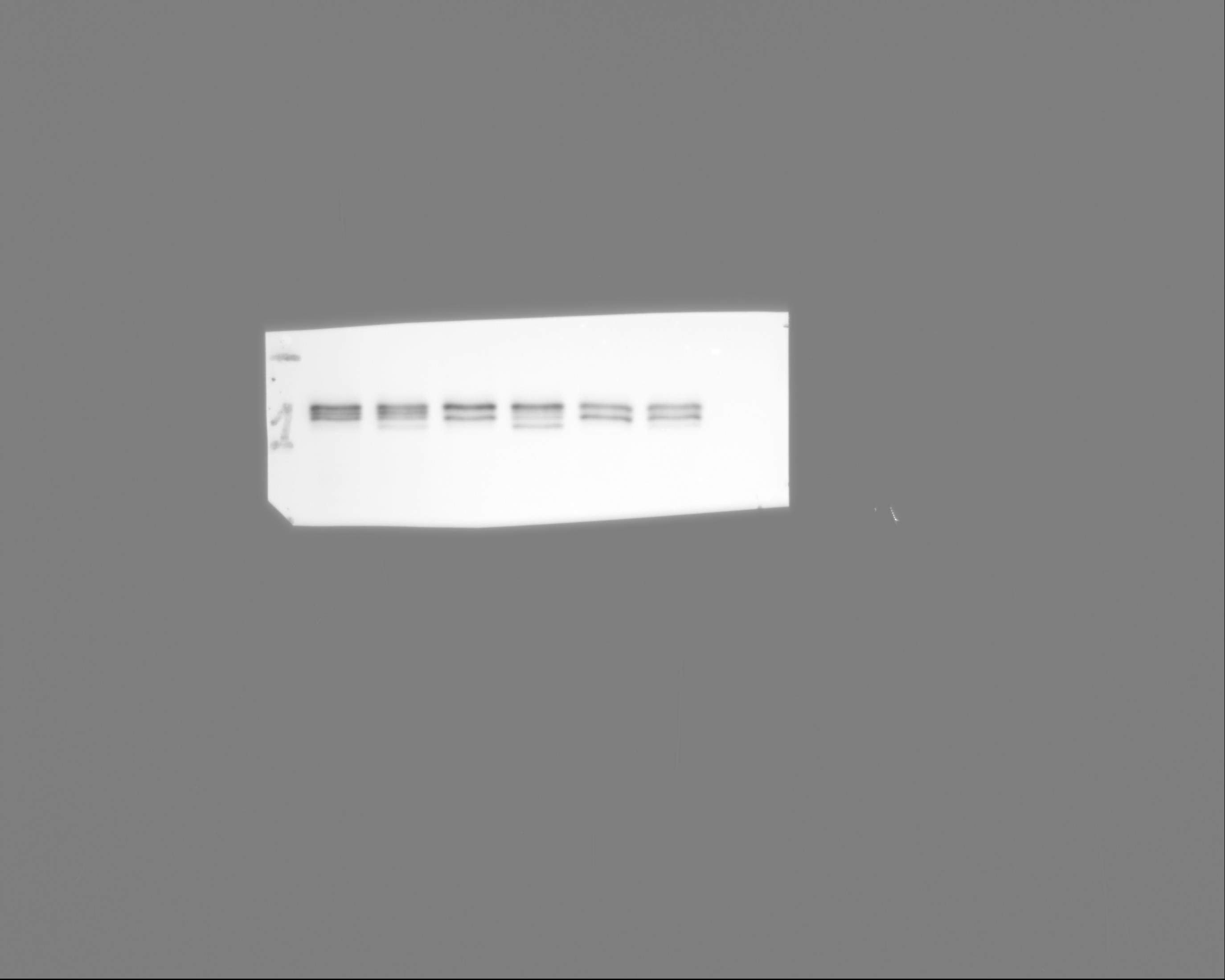

Supplement: Supplementary file 7 — Supplementary Material 7 [file 43440_2024_649_MOESM7_ESM.jpg]

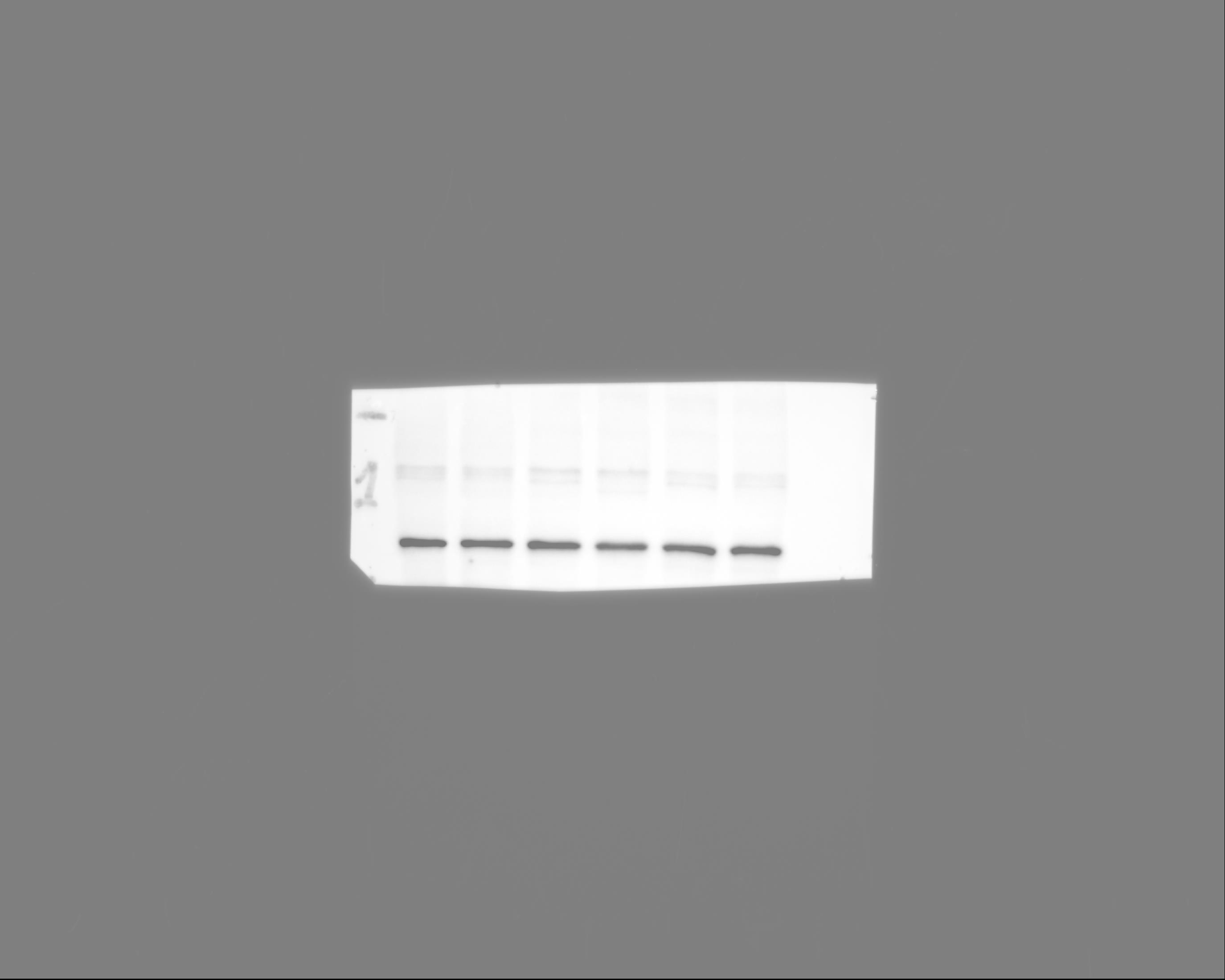

Supplement: Supplementary file 8 — Supplementary Material 8 [file 43440_2024_649_MOESM8_ESM.jpg]

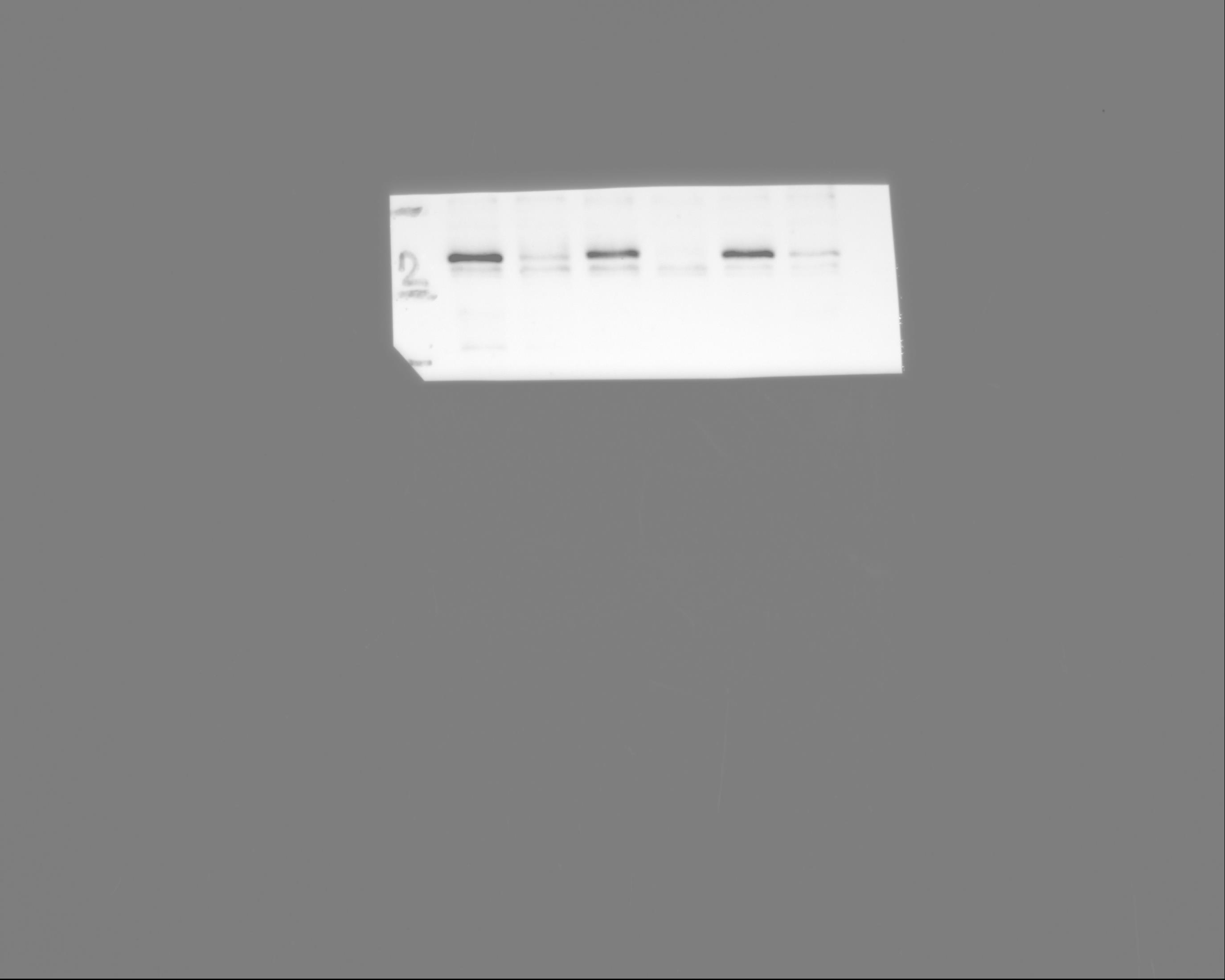

Supplement: Supplementary file 9 — Supplementary Material 9 [file 43440_2024_649_MOESM9_ESM.jpg]

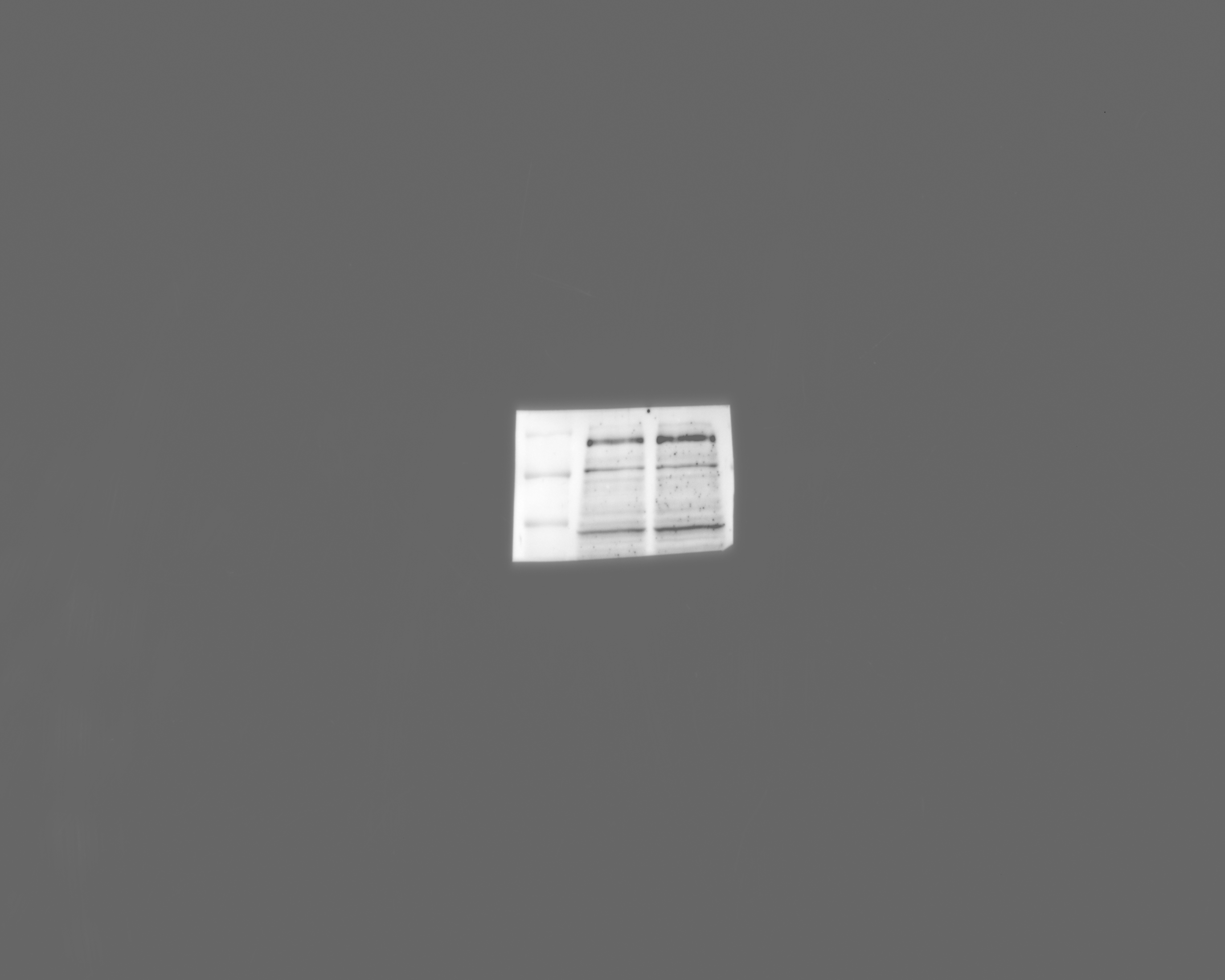

Supplement: Supplementary file 10 — Supplementary Material 10 [file 43440_2024_649_MOESM10_ESM.jpg]

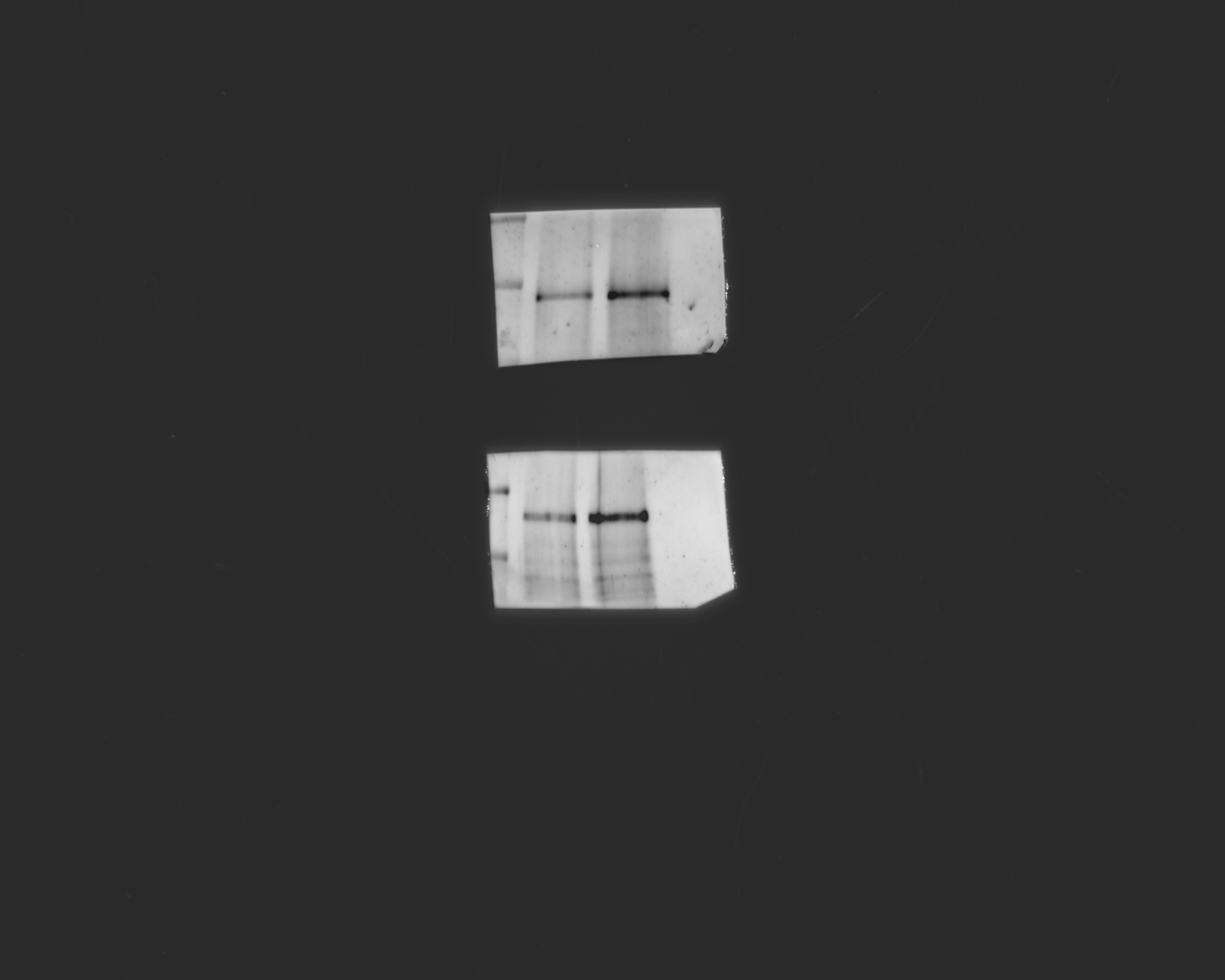

Supplement: Supplementary file 11 — Supplementary Material 11 [file 43440_2024_649_MOESM11_ESM.jpg]

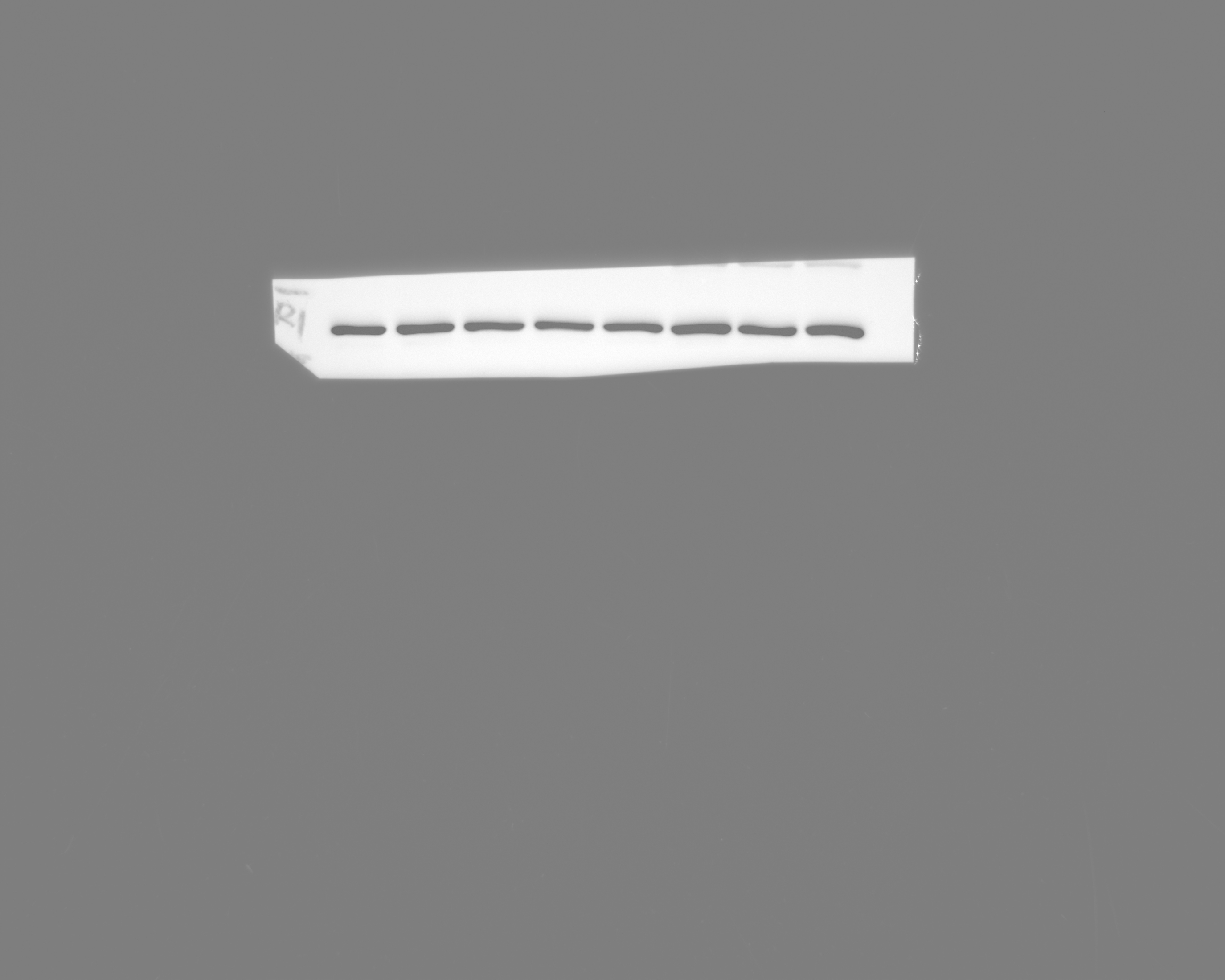

Supplement: Supplementary file 12 — Supplementary Material 12 [file 43440_2024_649_MOESM12_ESM.jpg]

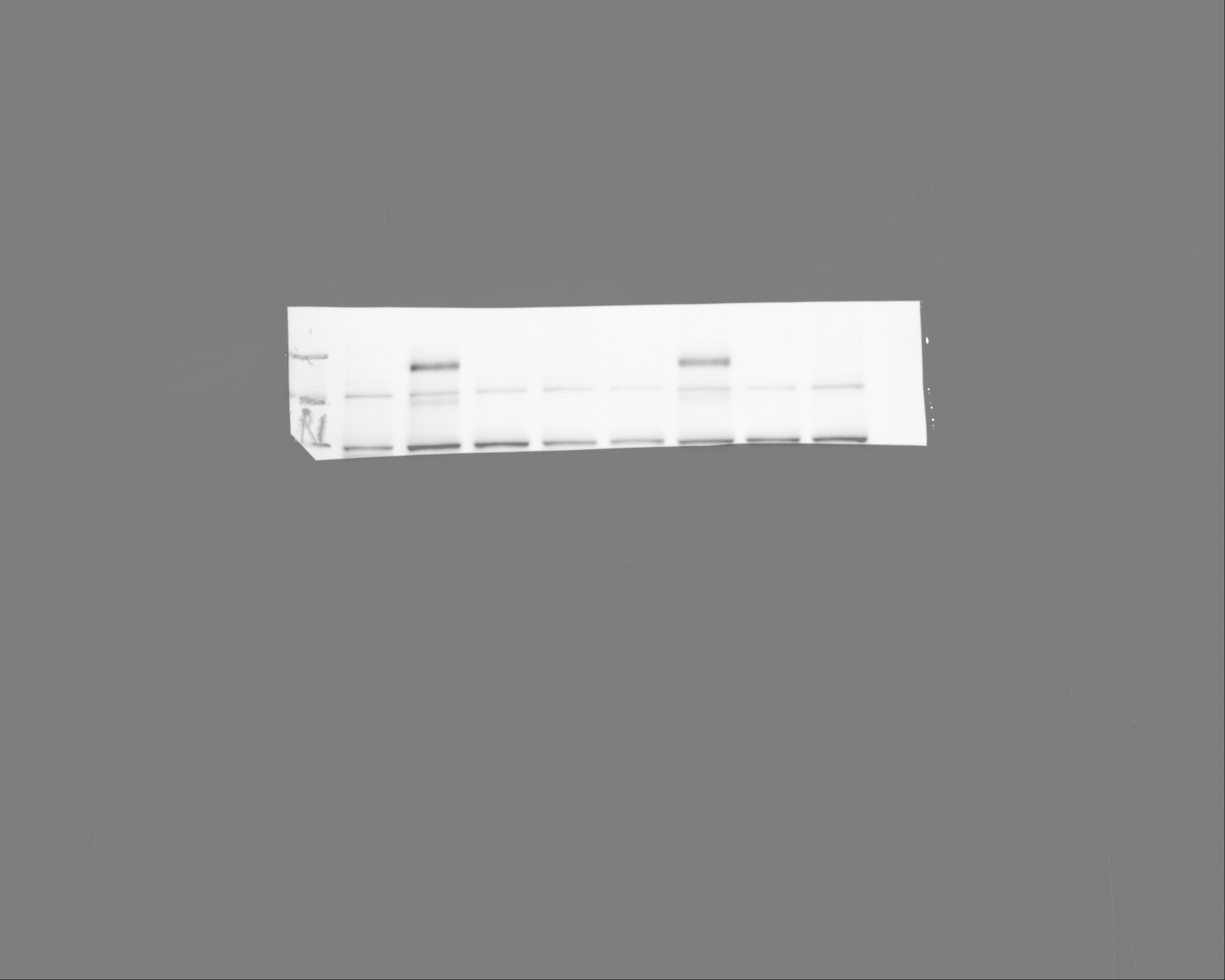

Supplement: Supplementary file 13 — Supplementary Material 13 [file 43440_2024_649_MOESM13_ESM.jpg]

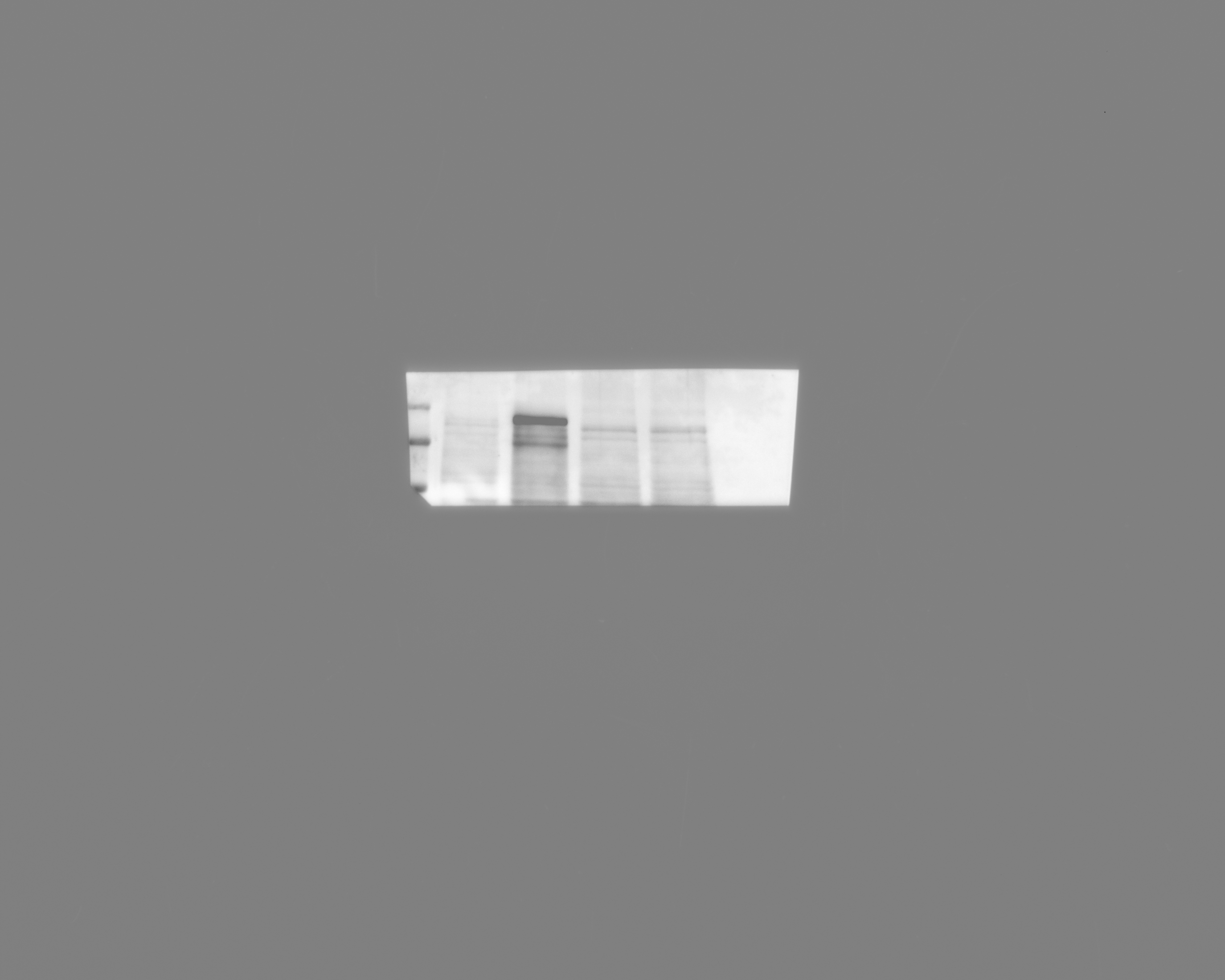

Supplement: Supplementary file 14 — Supplementary Material 14 [file 43440_2024_649_MOESM14_ESM.jpg]

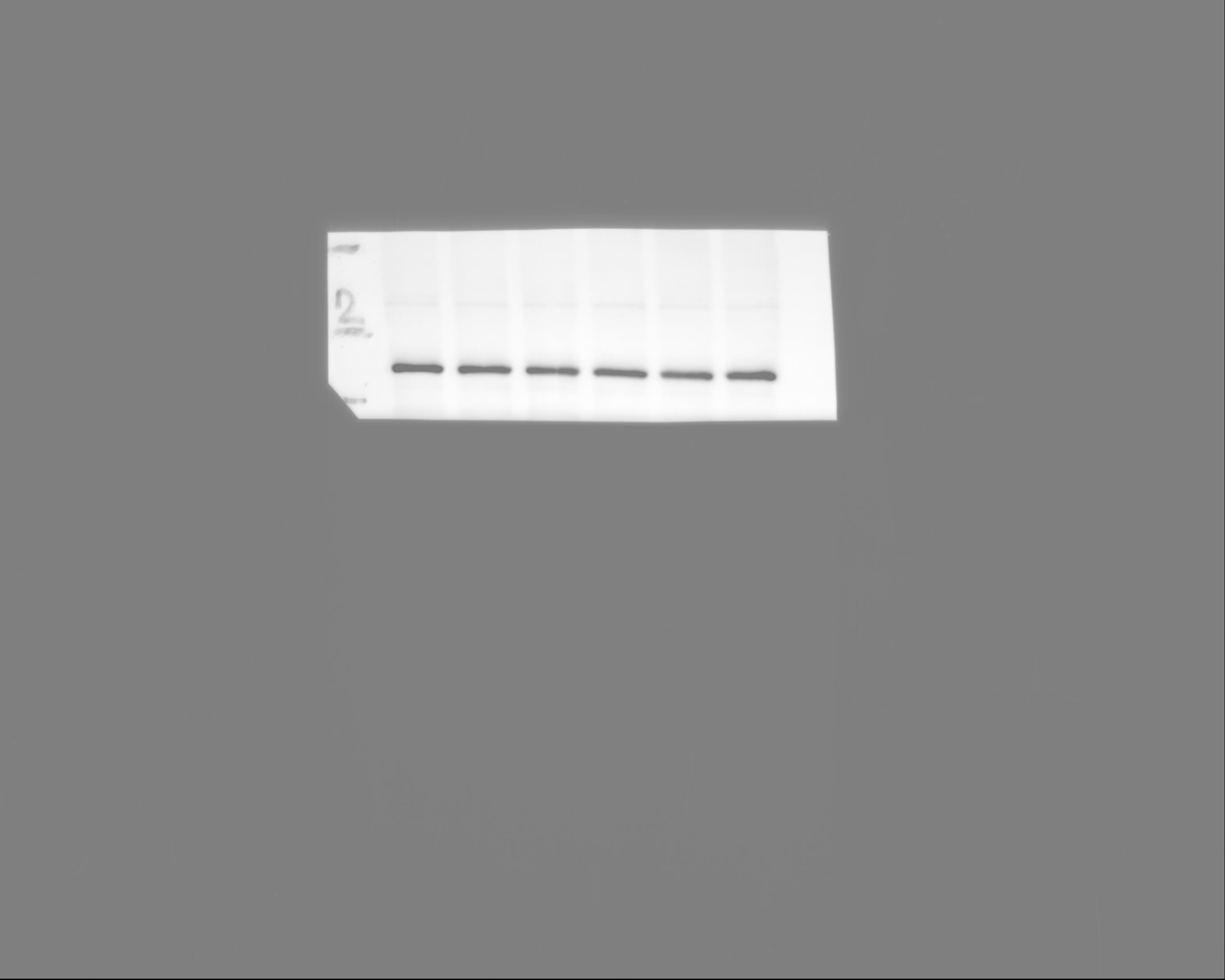

Supplement: Supplementary file 15 — Supplementary Material 15 [file 43440_2024_649_MOESM15_ESM.jpg]

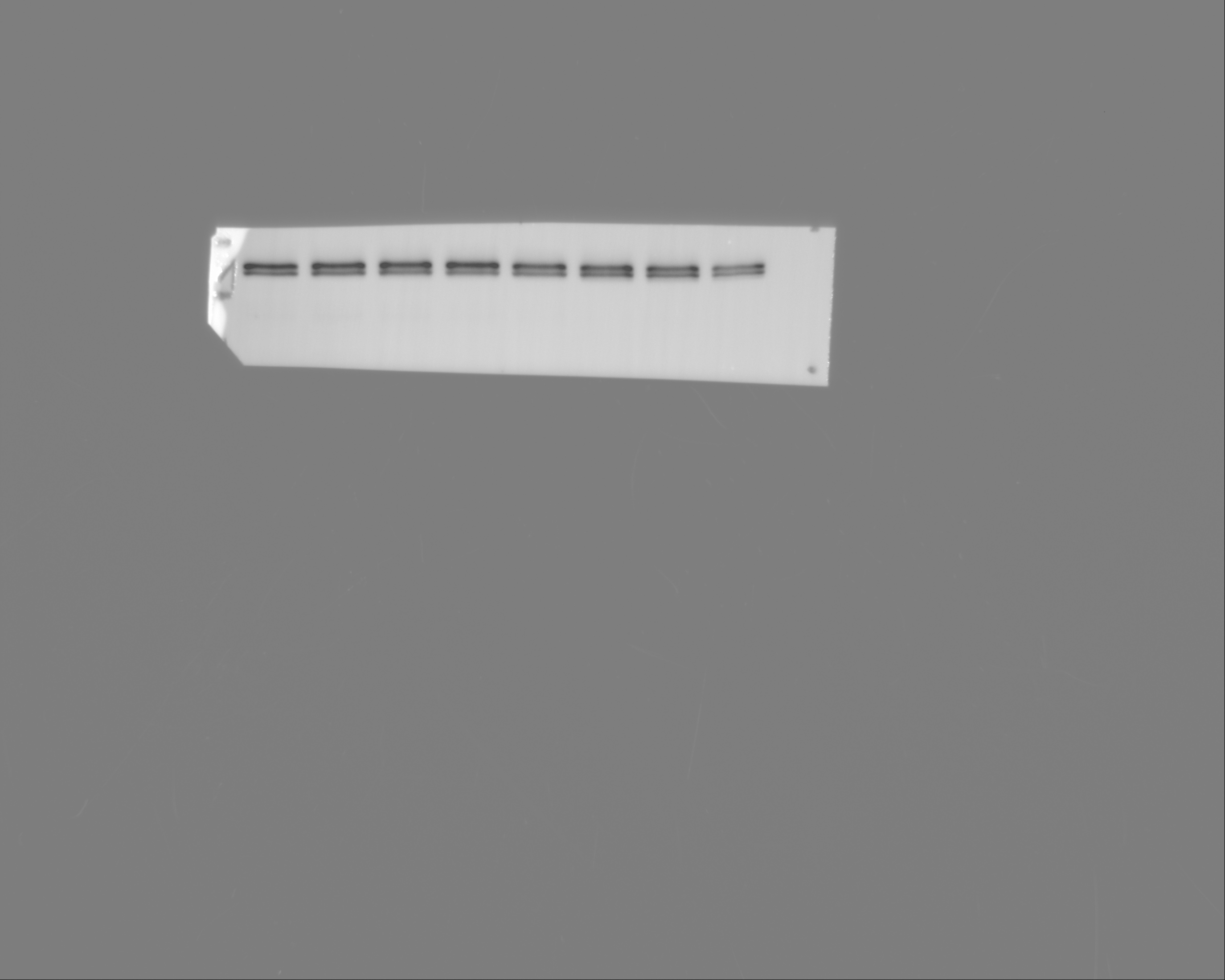

Supplement: Supplementary file 16 — Supplementary Material 16 [file 43440_2024_649_MOESM16_ESM.jpg]

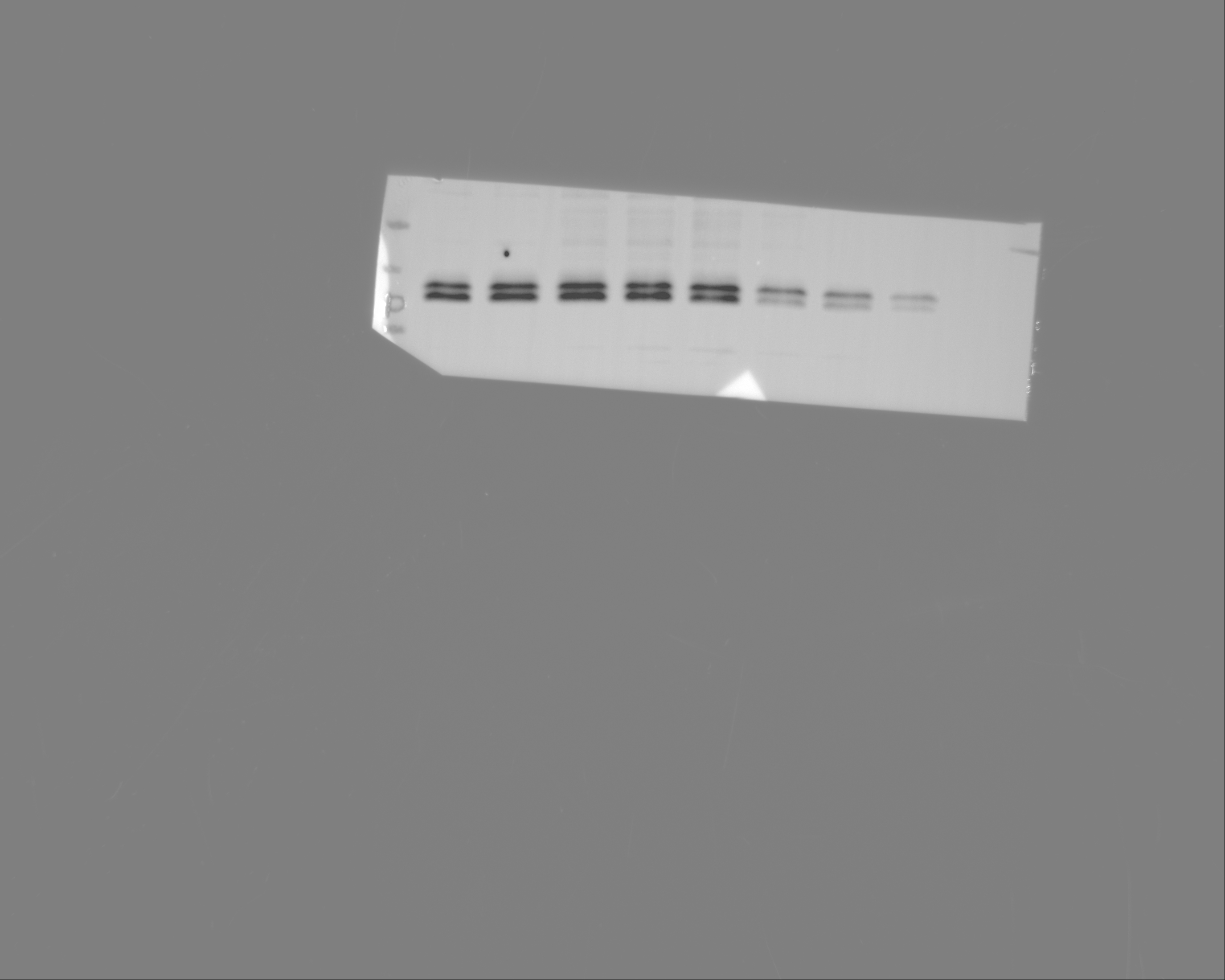

Supplement: Supplementary file 17 — Supplementary Material 17 [file 43440_2024_649_MOESM17_ESM.jpg]

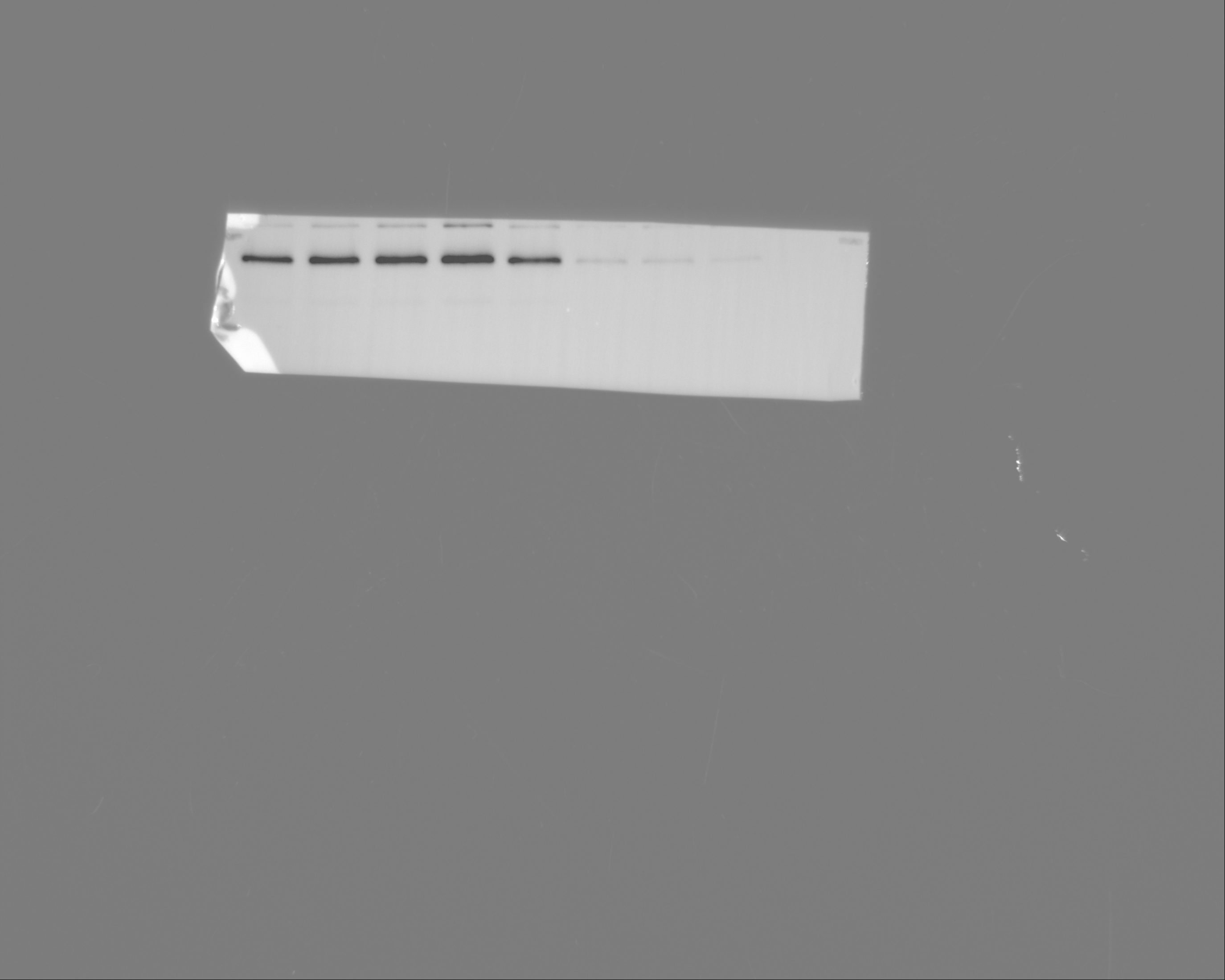

Supplement: Supplementary file 18 — Supplementary Material 18 [file 43440_2024_649_MOESM18_ESM.jpg]

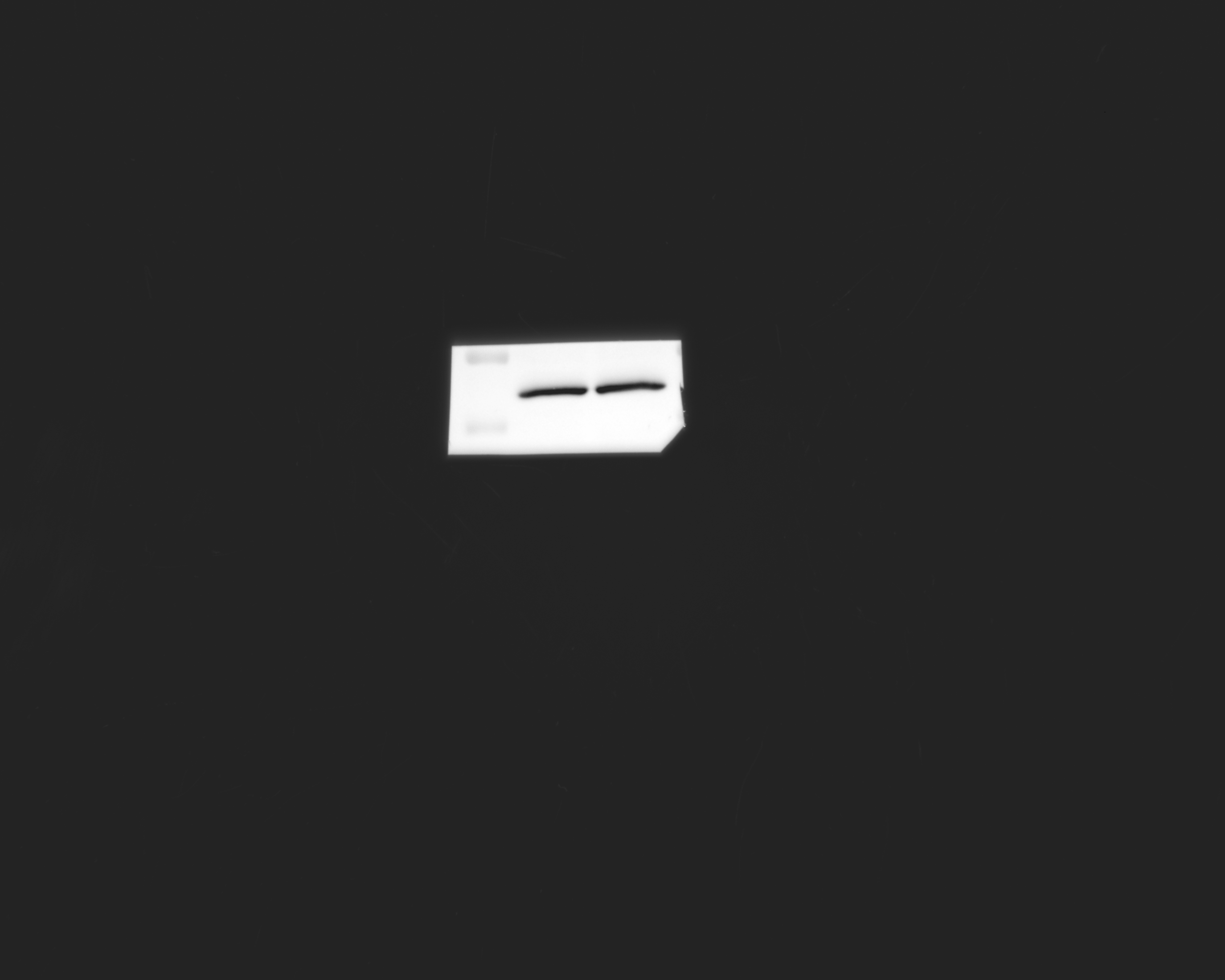

Supplement: Supplementary file 19 — Supplementary Material 19 [file 43440_2024_649_MOESM19_ESM.jpg]

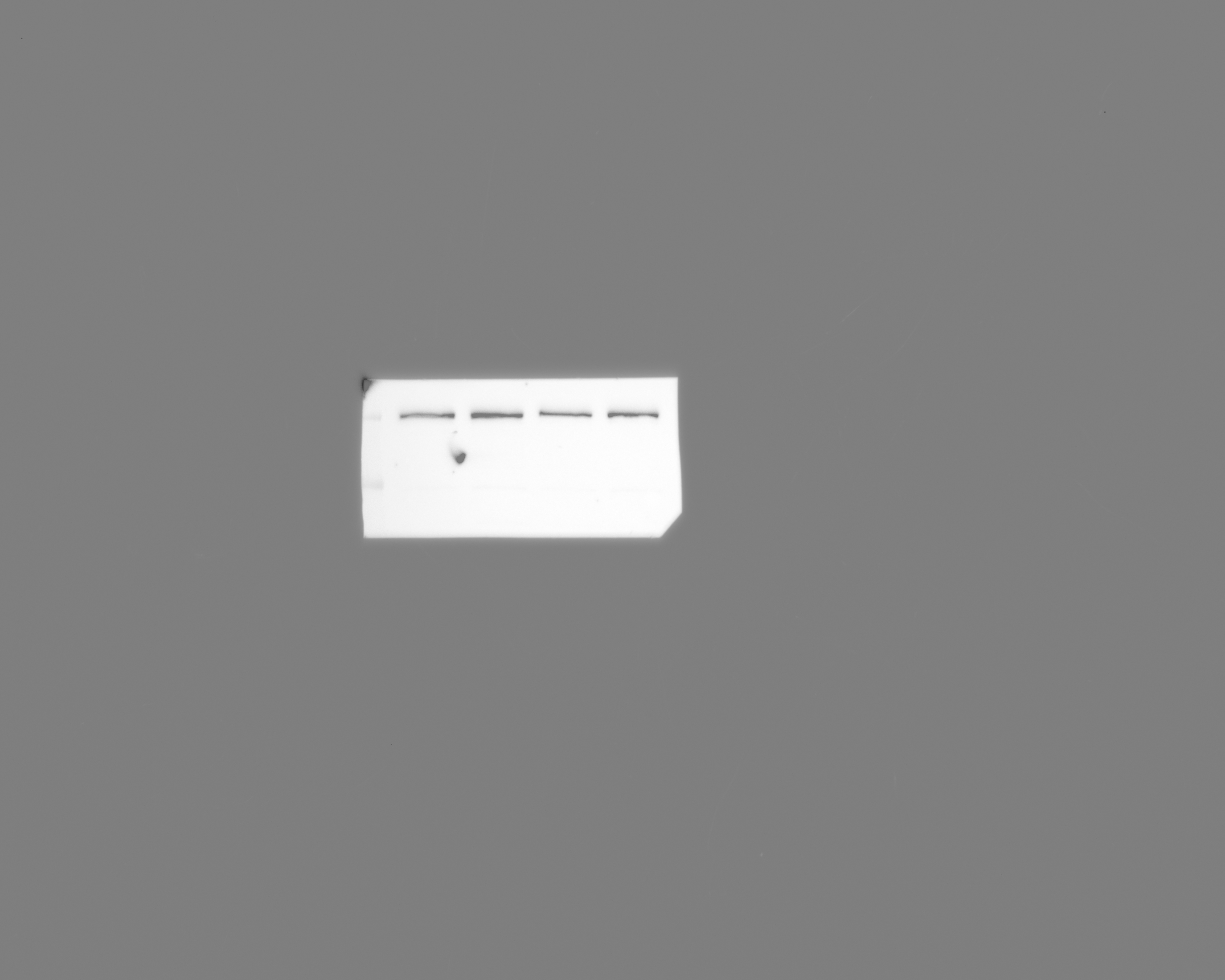

Supplement: Supplementary file 20 — Supplementary Material 20 [file 43440_2024_649_MOESM20_ESM.jpg]

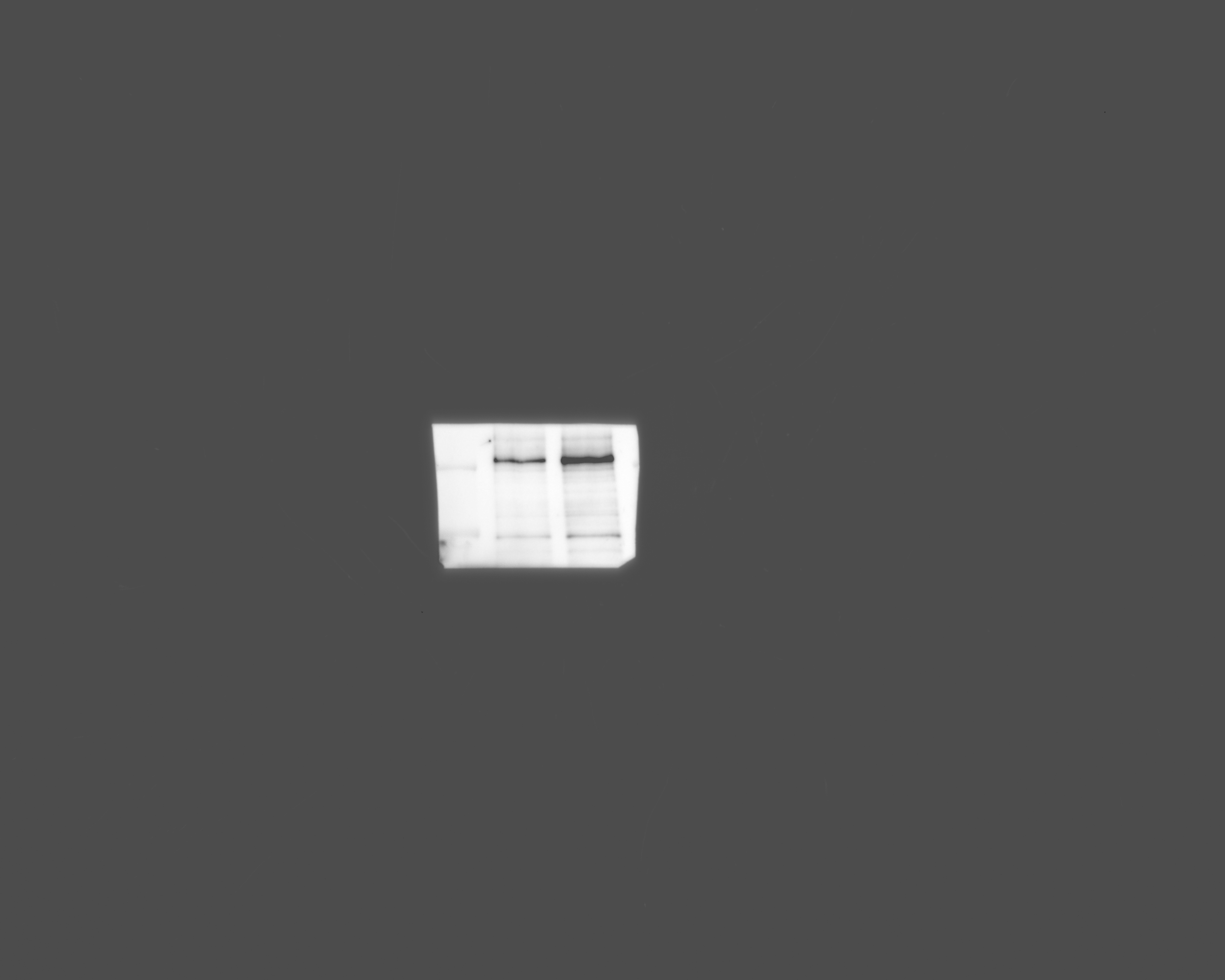

Supplement: Supplementary file 21 — Supplementary Material 21 [file 43440_2024_649_MOESM21_ESM.jpg]
